# Epigenomic and transcriptomic analyses reveal differences between low-grade inflammation and severe exhaustion in LPS-challenged murine monocytes

**DOI:** 10.1038/s42003-022-03035-2

**Published:** 2022-01-28

**Authors:** Lynette B. Naler, Yuan-Pang Hsieh, Shuo Geng, Zirui Zhou, Liwu Li, Chang Lu

**Affiliations:** 1 Department of Chemical Engineering, Virginia Tech, Blacksburg, VA USA; 2 Department of Biological Sciences, Virginia Tech, Blacksburg, VA USA

**Keywords:** Chronic inflammation, Epigenomics

## Abstract

Emerging studies suggest that monocytes can be trained by bacterial endotoxin to adopt distinct memory states ranging from low-grade inflammation to immune exhaustion. While low-grade inflammation may contribute to the pathogenesis of chronic diseases, exhausted monocytes with pathogenic and immune-suppressive characteristics may underlie the pathogenesis of polymicrobial sepsis including COVID-19. However, detailed processes by which the dynamic adaption of monocytes occur remain poorly understood. Here we exposed murine bone-marrow derived monocytes to chronic lipopolysaccharide (LPS) stimulation at low-dose or high-dose, as well as a PBS control. The cells were profiled for genome-wide H3K27ac modification and gene expression. The gene expression of TRAM-deficient and IRAK-M-deficient monocytes with LPS exposure was also analyzed. We discover that low-grade inflammation preferentially utilizes the TRAM-dependent pathway of TLR4 signaling, and induces the expression of interferon response genes. In contrast, high dose LPS uniquely upregulates exhaustion signatures with metabolic and proliferative pathways. The extensive differences in the epigenomic landscape between low-dose and high-dose conditions suggest the importance of epigenetic regulations in driving differential responses. Our data provide potential targets for future mechanistic or therapeutic studies.

## Abstract (web summary)

Lynette Naler and Yuan-Pang Hsieh et al. evaluate epigenomic and transcriptomic differences in mouse bone marrow-derived macrophages following exposure to high or low doses of LPS. Their results suggest that both low- and high-grade inflammation involve TRAM-dependent pathways.

## Introduction

Forming a close co-habitat with bacteria, mammalian hosts are intimately impacted by the presence of varying dosages of bacterial endotoxin lipopolysaccharide (LPS) within their circulation. While high-dose LPS triggers multi-organ injury and sepsis, through inducing pathogenic inflammation and immuno-suppression, lower dose LPS sustains a low-grade, or chronic inflammation, playing an important role in the onset or exacerbation of many chronic diseases, including Alzheimer’s disease^1^, depression^2^, diabetes^3^, and cancers^4^. Despite this, there still are no canonical markers for low-grade inflammation, partly due to a correlation of inflammatory activity with age^5,6^. Some recent studies in humans have attempted more sophisticated integrated approaches that show promise, but more research is required to identify key biomarkers and how they should be interpreted^6–8^.

Low-grade inflammation is often associated with certain lifestyle choices, like Western-style eating practices^9^, sedentary behavior^10,11^, sleep deprivation^12^, and social stress^13^. In fact, individuals who underwent major childhood stressors have elevated mortality and morbidity of immune or chronic diseases later in life, and epigenetic programming has been proposed^14^. This is further supported by twin studies that showed most variation in the immune system is due to non-heritable (i.e. epigenetic) influences^15^.

In a bacterial infection, components of the pathogen^16^ are recognized by pattern-recognition receptors (PRRs) that are found on immune and non-immune cells^17–20^. This, in turn, leads to a signaling cascade that is used to recruit immune cells, such as neutrophils and monocytes/macrophages, which ingest the microbes or release anti-microbials^21,22^. Once the threat is eliminated, pro-inflammatory macrophages uptake spent neutrophils, which reprogram the macrophages to an anti-inflammatory state^23,24^. Anti-inflammatory macrophages help promote healing by protecting against tissue damage, clearing out debris, and producing growth factors^24^. However, when macrophage and monocyte inflammatory activity is not resolved then it can lead to low-grade inflammation and chronic diseases^25–30^.

Lipopolysaccharide (LPS) is an endotoxin and component of the cell wall of Gram-negative bacteria, such as *Escherichia coli*, that also has been found at low levels in individuals who have chronic diseases or detrimental lifestyle choices as mentioned previously^31,32^. It has been used to study inflammation both in vivo and in vitro, although more often with high-doses of LPS. While persistent low-dose LPS has been shown to lead to a low-grade inflammatory state, the mechanism by which it does so is still not entirely known, though could be due to sustained activity of the inflammatory Nuclear Factor Kappa B (NF-κB)^32–35^.

LPS stimulation activates several different pathways, which both have pro- and anti-inflammatory aspects. First, LPS binds to Toll-like Receptor 4 (TLR4) and activates the Myeloid Differentiation Primary Response (MyD88)-dependent pathway^32,36,37^. The MyD88-dependent signaling cascade activates Mitogen-Activated Protein Kinases (MAPKs), NF-κB translocation to the nucleus, and transcription factors cAMP Response Element-Binding Protein (CREB) and Activator Protein 1 (AP-1), as well as induces expression of pro-inflammatory genes such as Tumor Necrosis Factor alpha (*Tnfa*), Interleukin 6 (*Il6*), and Prostaglandin-Endoperoxide Synthase 2 (*Ptgs2*)^37^. However, it is also involved in the production of anti-inflammatory cytokines like Interleukin 10 (IL10). In addition, MyD88-dependent signaling leads to an increase in glycolysis, synthesis of acetyl-CoA, and synthesis of fatty acids. Next, TLR4 can also trigger the Translocation Associated Membrane Protein (TRAM)/TIR-domain-containing adapter-inducing interferon-β (TRIF)-dependent pathway^37^. The TRIF-dependent pathway activates Interferon Regulatory Factor 3 (IRF3) and Interferon Regulatory Factor 7 (IRF7) to induce expression of type I interferons (IFNs), C-C Motif Chemokine Ligand 5 (*Ccl5*), and C-X-C Motif Chemokine Ligand 10 (*Cxcl10*). It is also involved in anti-inflammatory cytokine IL10 production and is important for *Tnfa* expression^37,38^. Furthermore, in macrophages, perhaps independent of TRAM, the TRIF-mediated pathway also upregulates cell-surface Cluster of Differentiation 40 (*Cd40*), Cluster of Differentiation 80 (*Cd80*), and Cluster of Differentiation 86 (*Cd86*) which are necessary for antigen presentation for T lymphocytes, bridging the gap between the innate and adaptive immune system^37,39^. The exact role of TRAM, however, is not well understood in the cellular responses to either low or high doses of LPS. Finally, both pathways are involved in the canonical activation of the NLR Family Pyrin Domain Containing 3 (NRLP3) inflammasome, which is responsible for the activation of the inflammatory cytokine, Interleukin 1 beta (IL-1β), and can cause pyroptosis, a form of programmed cell-death^37^.

The epigenome plays a large role in the behavior and identity of macrophages. For example, the epigenome of tissue-resident macrophages is affected by their local microenvironment and they can even be reprogrammed by transplanting them to a different location^40^. They also have varied transcriptional signatures during efferocytosis, a process where apoptotic cells are cleared^41^. LPS-stimulation has also been shown to have a significant, and sometimes lasting, effect on the histone modifications of macrophages^42^. LPS-induced tolerance affects the epigenome of macrophages by inhibiting induction of inflammatory genes while leaving antimicrobial genes unaffected^43^. Research into means of targeting and altering the epigenome of inflammatory macrophages into an anti-inflammatory state has also increased in recent years, with studies showing possible therapeutic potential^44^. Despite this, there is little research on how low-doses of LPS affect the epigenome^45–48^ or transcriptome^48–52^ of monocytes or macrophages differently compared to high-doses of LPS.

In this study, we profiled the histone mark H3K27ac and performed RNA-seq analysis of murine bone marrow-derived monocytes that are exposed to low and high-dose levels of LPS, as would be seen in low-grade inflammation and immune exhaustion respectively. Low-input technologies including Microfluidic Oscillatory Washing ChIP-seq (MOWChIP-seq^53,54^) and Smart-seq2^55,56^ were used for the epigenomic and transcriptomic profiling, respectively. We compared the conditions to extract the effects of LPS-dosage on the epigenome and, in turn, the transcriptome of immune cells. Furthermore, we also analyzed the effects of *Tram* deletion, in order to clarify the role of this less-studied TLR4 adapter, as well as Interleukin 1 Receptor Associated Kinase M (*Irak-m*) deletion. Although IRAK-M is a known inhibitor of TLR4 pathway, the exact role of IRAK-M in modulation of low-grade inflammation and monocyte exhaustion is not well defined^57^. We revealed that TRAM is at least partially involved in the generation of both low-grade inflammatory monocytes, as well as the development of exhaustion, through facilitating Signal Transducer and Activator of Transcription 1 (STAT1) / IRF7 activation and induction of selective exhaustion markers such as S100 Calcium Binding Protein A8 (S100A8), often seen in patients with COVID-19. The impacts of *Irak-m* deletion are more nuanced, by exacerbating low-grade inflammation and refraining the development of exhaustion. Altogether, our analyses provide population-level adaptation of monocytes to the persistent challenges of LPS with varying signal strengths.

## Results

### TRAM facilitates inflammatory monocyte generation under both low-dose and high-dose LPS conditions

**Fig. 1:**
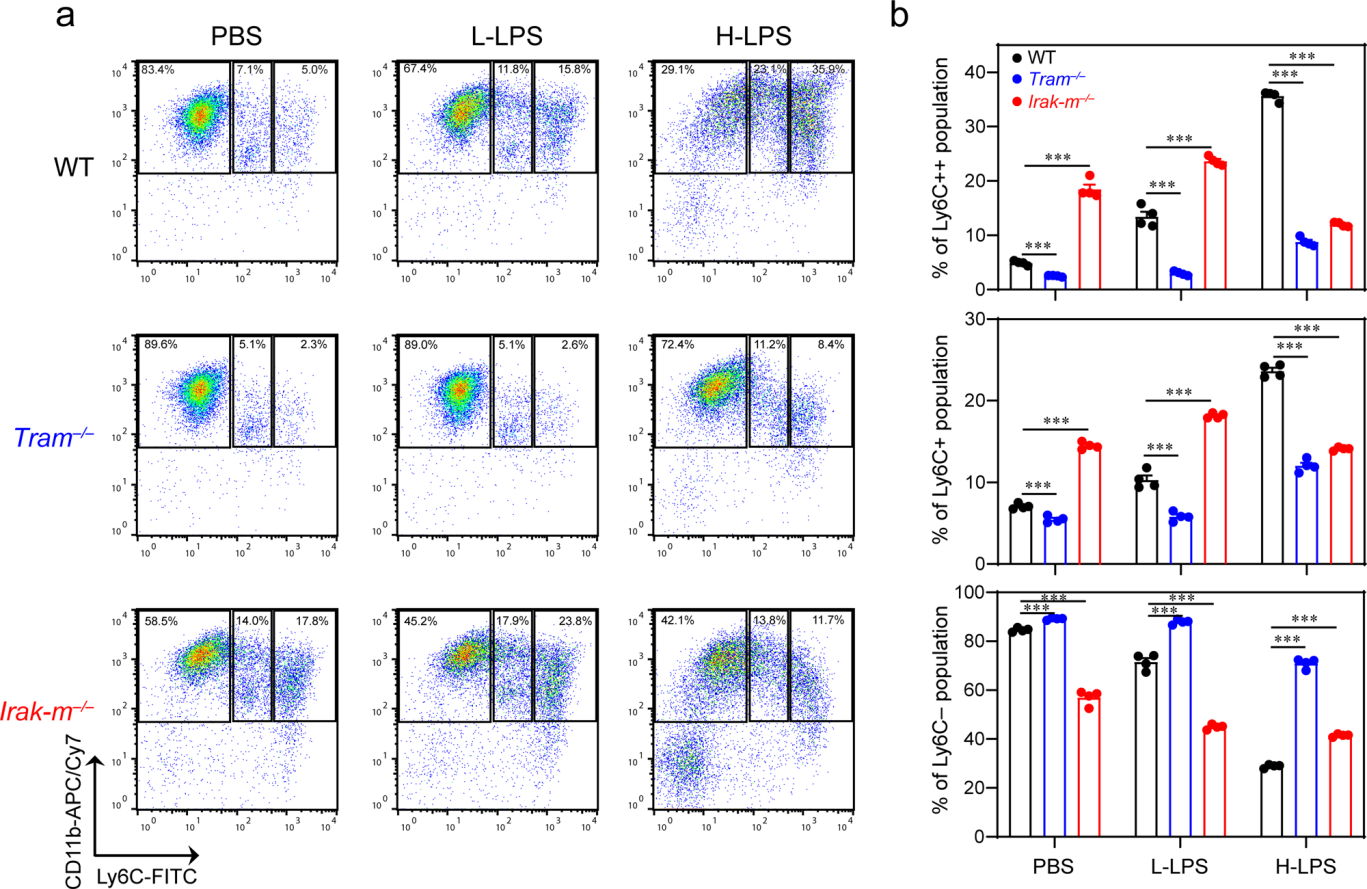
The generation of inflammatory monocytes under persistent low-grade or exhaustive challenges, facilitated by TRAM and finely modulated by IRAK-M. Primary murine monocytes from WT, *Tram*^−/−^ and *Irak-m*^−/−^ mice were treated with either PBS, low-dose LPS (100 pg/mL) or high-dose LPS (1 µg/mL) for 5 days, and monocyte subsets were analyzed with flow cytometry. **a** Representative staining profile of the monocyte subsets. **b** Quantification of the populations of non-classical (Ly6C−); intermediate (Ly6C+); and classical monocytes (Ly6 + +). Data are representative of three independent experiments, and error bars represent means ± SEM. ***P < 0.001; one-way ANOVA (*n* = 4 for each group).

Bone marrow-derived monocytes (BMDMs) were isolated from mice (Suppl. Fig. 1) and dosed with PBS, low-dose LPS (100 pg/mL), or high-dose LPS (1 µg/mL) for a 5 day period as we performed previously^58,59^. Persistent challenges with low-dose LPS enabled the generation of inflammatory monocytes (both the intermediate Ly6C + and the classical Ly6C++ monocytes) (Fig. 1), consistent with our previous reports^58,60^. In contrast, exhaustive stimulations with high-dose LPS almost completely depleted the Ly6C- non-classical monocytes, and led to a drastic expansion of the Ly6C++ monocytes (Fig. 1a). The sharp reduction of non-classical monocytes was also observed in septic patients including COVID-19 patients, suggesting the development of innate immune exhaustion^61–64^. However, the detailed profiles and underlying mechanisms of both low-grade inflammatory monocytes and exhausted monocytes are not well defined. Given our previous findings that TRAM is involved in the generation of low-grade inflammatory monocytes^33,60,65^, we then evaluated the development of both low-grade inflammation (with low-dose LPS) and exhaustive inflammation (with persistent challenges of high-dose LPS) comparing WT and TRAM-deficient monocytes. Indeed, we observed that the expansion of Ly6C+ and Ly6C++  inflammatory monocytes were completely ablated in TRAM-deficient monocytes when challenged with low-dose LPS (Fig. 1b, Suppl. Data 1). Interestingly, the drastic depletion of non-classical Ly6C- monocytes and severe expansion of exhausted monocytes seen in WT cells challenged with high dose LPS were also attenuated in TRAM-deficient monocytes, suggesting a role of TRAM in both events. We also examined the involvement of IRAK-M during the generation of low-grade vs exhausted monocytes. Consistent with previous findings, we observed that *Irak-m* deletion enabled the significant expansion of Ly6C+ and Ly6C++  inflammatory monocytes under low-grade inflammatory condition (Fig. 1b). In contrast, *Irak-m* deletion attenuated the drastic expansion of Ly6C+ and Ly6C++  inflammatory monocytes under the exhausted high-dose LPS challenge. Our data suggest that TRAM may function as a gate-keeper for sensing LPS challenge, while IRAK-M may serve as a downstream fine tuner for the adaption of monocytes.

### Higher LPS dosage causes larger overall change in the epigenome

**Fig. 2:**
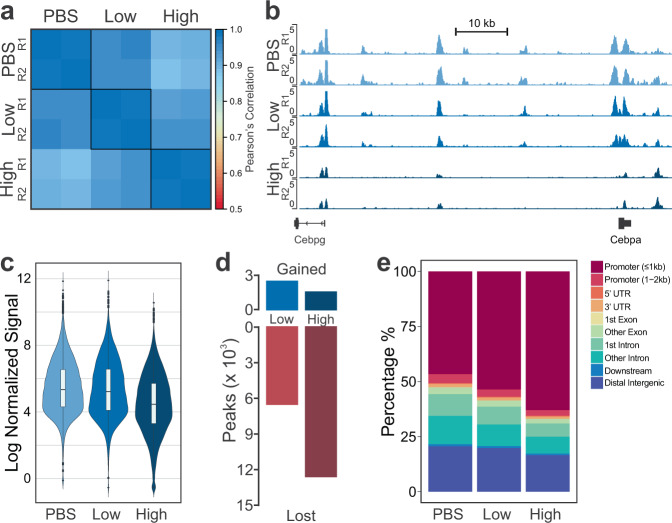
Overview of epigenomic data for murine BMDMs dosed with PBS, low-dose LPS, or high-dose LPS. **a** Pearson’s correlation of normalized H3K27ac signal around promoter regions (TSS ± 2 kb). **b** Tracks of normalized H3K27ac signal for replicates dosed with PBS, low-dose LPS, or high-dose LPS (from top to bottom). Tracks are aligned in mm10 and the region displayed is chr7:35,042,108-35,138,618. **c** Distribution of normalized H3K27ac signal at peaks (*n* = 29,071). The middle bar of the boxplot denotes the median. The upper and lower bounds of the box correspond to the 75th and 25th percentile, respectively. Each whisker spans up to the range of 1.5 * interquartile range from the edge of the box. **d** Number of peaks gained or lost in low-dose or high-dose conditions compared to PBS. **e** Percentage of peaks at genomic locations.

To characterize the global profiles of epigenetic alterations, we then profiled H3K27ac using MOWChIP-seq^54^ and performed RNA-seq with two replicates per condition (Suppl. Tables 1 and 2). We found very high average correlation between ChIP-seq replicates of 0.986 (Fig. 2a). Between conditions, the highest correlation is between PBS and low-dose (0.957) and the lowest correlation between PBS and high-dose (0.896), with low-dose and high-dose falling in-between (0.939). This suggests that the increasing LPS dosage causes a concomitant change to H3K27ac signal. When looking at genome-wide H3K27ac signal, we see that increasing the dose of LPS tends to reduce the H3K27ac signal (Fig. 2b). In fact, the overall H3K27ac signal at peaks also tends to go down with increasing LPS-dosage (Fig. 2c, Suppl. Data 2). Furthermore, the number of peaks present in each sample was decreased with increasing dosage (PBS  =  25,679, Low = 21,597, High = 14,659). As such, many of the peaks that were present in PBS samples were not present in low-dose samples (6,566) and even more (12,602) were not present in high-dose (Fig. 2d, Suppl. Data 2). However, low-dose samples gained a small number of peaks (2,484) while high-dose samples gained even fewer (1,582). In addition, the fraction of peaks near promoters increases with increasing LPS dosage, yet this primarily was due to a reduction of distal peaks, rather than an increase of proximal peaks (Fig. 2e, Suppl. Data 2). However, some of the peaks gained by low-dose and high-dose conditions were proximal to gene promoters. Since H3K27ac also localizes to active promoters, this suggests that LPS dosage is activating genes that are not active under normal conditions^66^.

### Low-dose and high-dose LPS stimulations have different effects on enhancers

**Fig. 3:**
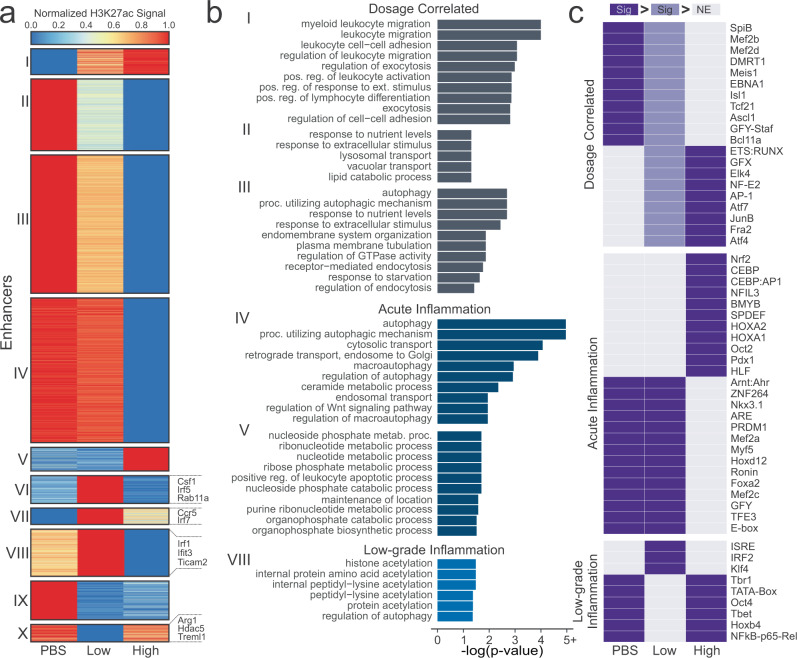
Effect of LPS dosage on enhancers. **a** K-means clustering of enhancers present in PBS, Low-dose, or High-dose samples. **b** Significant (FDR < 0.05) Gene Ontology biological process gene sets for clusters from (**a**). **c** Motifs that are significantly enriched (*p* < 1 × 10^−6^) in at least one condition compared to another. Dark purple cells denote that motif is significantly enriched (*p* < 1 × 10^−6^) in that experimental condition in relation to any light purple or gray cells. Light purple cells are used as a middle ground such that dark purple is more significant than light purple, and light purple is more significant than gray. Non-enriched gray cells (NE) do not have a *p*-value cutoff, as they refer to the conditions with the lowest enrichment of that motif.

Although H3K27ac does mark active transcription, it plays a more pivotal role in long-range gene regulation at enhancer regions. Therefore, we determined the locations of active enhancers, which were defined as H3K27ac^high^ regions that do not overlap with regions near transcription start sites (TSS) and can be linked to genes in conjunction with RNA-seq data (see Methods). Thus, each of the active enhancers we describe have a statistically significant link to their gene. As these active enhancers very likely regulate their linked genes, we use these genes to determine significantly enriched pathways. This allows us to leverage and integrate our epigenomic and transcriptomic data. For simplicity, we will refer to these active enhancers simply as ‘enhancers’. The number of enhancers also decreased with increasing LPS-dosage (PBS  =  5,738, Low = 4,400, High = 2,711). Normalized H3K27ac signal for the conditions at each enhancer was clustered with k-means clustering (Fig. 3a, Suppl. Data 3). The genes linked to the enhancers in each of the clusters were analyzed for overrepresentation of Gene Ontology biological process gene sets (Fig. 3b, Suppl. Data 3). We then separated these clusters into three groups: dosage correlated (I, II, III), acute inflammation (IV and V) and low-grade inflammation (VIII).

The dosage correlated group, including clusters I, II, and III, had either increased or decreased H3K27ac signal with increased LPS-dosage. Most obviously, we see that Cluster I, which increases in expression from PBS to high-dose, is filled with leukocyte-related (which include monocytes and macrophages) gene ontologies, which play a key role in inflammation^67,68^. As such, it is reasonable that with increased LPS-dosage we would see a stronger inflammatory response. In Clusters II and III, which decreased from PBS to high-dose, we see several pathways related to autophagy and endocytosis. It has been shown in literature that LPS-stimulation induces autophagy as a means of modulating the inflammatory response^69–72^. Upon further investigation into the enhancer-linked genes in these autophagy-related processes, we find genes such as *Trem2*^73^, *Sesn1*^74^, and *Nrbf2*^75^, all of which inhibit the inflammatory effect of macrophages. Differential H3K27ac expression at gene promoters also shows that increased LPS-dosage reduces expression at genes that negatively regulate components of the TLR4 signaling pathway, such as the MAPK cascade (Suppl. Fig. 2, Cluster III)^37,76,77^.

The exhausted group, where the signal was either substantially lower or higher in high-dose LPS compared to low-dose LPS and PBS, consists of clusters IV and V. These enhancers are recognized as highly characteristic of the high-dose condition and associated innate exhaustion. In Cluster IV, we once again see autophagy-related pathways. Gene promoters with substantially lower H3K27ac signal in high-dose cells were enriched in pathways for IL6 and IL8 production, which can have pathogenic inflammatory and immuno-suppressive effects characteristic of exhausted monocytes (Suppl. Fig. 2, Cluster IV)^76,77^. In Cluster V, we see many nucleotide-associated metabolic and catabolic processes, which have been shown to be increased with LPS stimulation in literature^78,79^. It is unclear why these processes are not increased in low-dose cells.

The low-grade inflammation group, in which signal was either substantially increased or decreased in low-dose LPS when compared to high-dose LPS or PBS, consists of clusters VI, VII, VIII, IX, and X. These enhancers are recognized as highly characteristic of the low-dose condition and associated low-grade inflammation. Only Cluster VIII had any significantly enriched terms, most of which were related to histone and protein acetylation, primarily acetylation at lysines. LPS stimulation has been shown in literature to affect histone acetylation^80^, but we do not have enough information to determine which modifications (other than H3K27ac) are affected uniquely by low-dose LPS, though aberrant histone acetylation has been found in multiple chronic inflammatory diseases^81,82^. However, the enhancers within this grouping were linked to some interesting genes within the immune system. For example, enhancers who had much higher signal in low-dose were linked to genes like *Csf1*, *Irf5*, *Rab11a*, *Ccr5*, *Irf7*, *Ticam2*, *Ifit3*, and *Irf1*, while enhancers with lower signal in low-dose were linked to genes like *Arg1*, *Hdac5*, and *Treml1*. RAB11A is responsible for transporting TLR4 from the endocytic recycling compartment to forming phagosomes^37^. This transport triggers the TRIF-dependent signaling pathway, which requires TRAM (*Ticam2*). TRAM/TRIF-dependent signaling leads to increases in interferon-alpha and interferon-beta production which, in turn, activate interferon-induced genes such as *Ifit3*. *Hdac5*^83^ and *Treml1*^84^ both regulate the inflammatory response. Furthermore, reduction of *Hdac5* expression was also associated with an increase of *Irf1* and transcription of interferon-beta^83^. In addition, *Arg1*, which is a typical marker of anti-inflammatory macrophages^49^, is reduced in low-dose while *Irf5*, shown to promote pro-inflammatory macrophage polarization^85^, is increased in low-dose. Together, these suggest that, while the low-dose cells are markedly different from high-dose cells, they do have a pro-inflammatory phenotype, which is consistent with previous studies^33^. It is interesting to note that *Csf1*, which is associated with anti-inflammatory macrophages, is highly increased in enhancer signal in low-dose cells, however, previous studies have shown an association between *Csf1* and chronic inflammation^86^.

Since enhancers are hotbeds of transcription factor binding activity, the enhancer regions were then scanned for transcription factor binding motifs that were separated into motifs uniquely and significantly enriched or diminished in low-grade inflammation, acute inflammation, or if the enrichment was correlated to LPS-dosage (Fig. 3c). Among the dosage-dependent affected motifs, we see multiple immune-related transcription factors that are increased with increasing LPS-stimulated inflammation such as JUNB^87^, AP-1^88,89^, ATF4^90^, and NFEE2^51^.

In exhaustive inflammation, we see several transcription factors that are important in the TLR-signaling pathway such as NFIL3^91,92^, HOXA2^93^, NRF2^94^, CEBP:AP1^95,96^, and OCT2^97,98^, all of which are activated with LPS stimulation in literature. In fact, the differential enrichment of NRF2 between high-dose and low-dose is also supported by previous research. NRF2 is activated by LPS stimulation via the reduction of the protein KEAP1, however, KEAP1 protein has been shown to accumulate in low-dose conditions^33,94^. The MEF2 family (MEF2A, MEF2B, MEF2C, MEF2D) of transcription factors motifs are significantly deficient in high-dose cells, but are present in low-dose cells. It has been show that the MEF2 family is initially upregulated by LPS-stimulation but are soon downregulated^51^. It is possible that, in the low-dose condition, the LPS-stimulation is not enough to lead to the downregulation of the MEF2 family.

Low-grade inflammation leads to fewer enriched motifs than deficient motifs. Motifs that are enriched only in low-dose are ISRE and IRF2. IRF2^99,100^ is an inflammation regulator while ISRE is an IFN-I stimulating response element which, when bound, activates genes in the inflammation pathway^101^. This is consistent with previously discussed upregulated enhancer-linked genes. Additionally, IRF2 has been shown to positively regulate the non-canonical inflammasome pathway, which, in turn, leads to increased *Gsdmd* expression^102^. In fact, we do see increased *Gsdmd* expression in our enhancer data, where it is located in Cluster VI. Low-dose also has reduced P65 motifs, a NF-κB subunit that is part of the canonical pathway involved in inflammation^103^. However, in monocytic cells that have already been stimulated with a Gram-negative bacteria, a second stimulation shows reduced P65 activity^104,105^. There is a possibility that sustained low-dosage of LPS may lead to such a reaction. Furthermore, it appears that reduced P65 expression can be somewhat compensated for^106^.

### *Tram−/−* has more profound effects on enhancer activity than *Irak-m−/−*

**Fig. 4:**
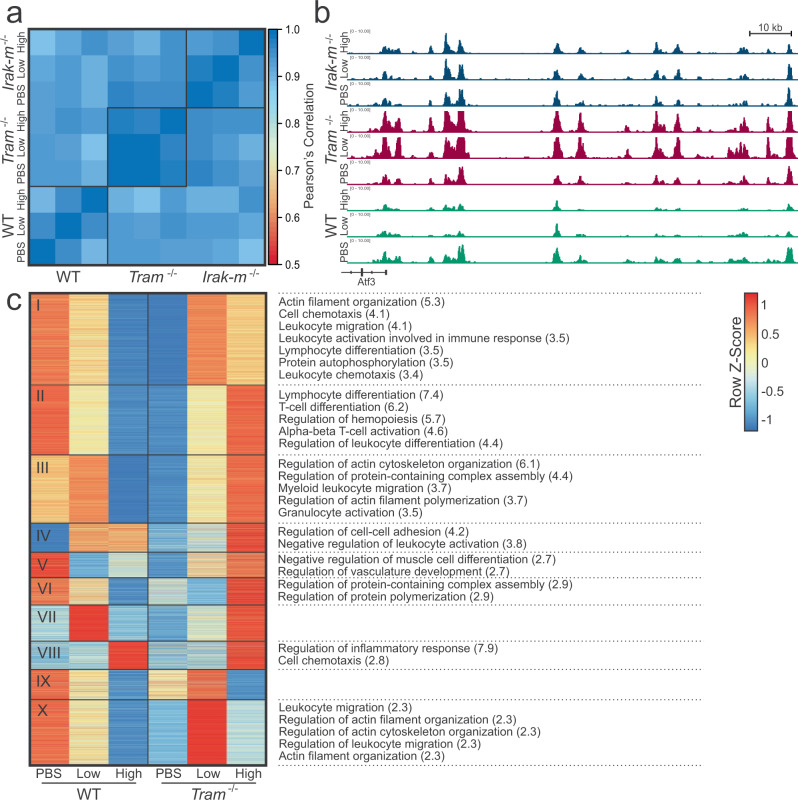
Effect of LPS on H3K27ac in TRAM-deficient and IRAK-M-deficient cells. **a** Pearson’s correlation of normalized H3K27ac signal at promoter regions (TSS ± 2 kb) of WT, *Tram*−/−, and *Irak-m*−/−. **b** Representative tracks of normalized H3K27ac signal from WT, *Tram*−/−, and *Irak-m*−/−. Tracks are aligned in mm10 and the region displayed is chr1:191,173,497-191,334,210. **c** Normalized H3K27ac signal at WT enhancers in WT and TRAM-deficient cells. Significant biological process gene ontologies (FDR < 0.05) listed on the right, with log(FDR) in parentheses.

Next, we analyzed how TRAM-deficiency and IRAK-M deficiency alters the effects of increasing LPS dosage on H3K27ac signal and enhancer activity (Fig. 4). Similar to WT, the PBS and low-dose conditions for both TRAM-deficient and IRAK-deficient cells were more correlated with one another (0.990 and 0.980, respectively) than with the high-dose condition (average 0.967 and 0.943, respectively), though the differences were small (Fig. 4a). Correlation among the experimental conditions (PBS  =  0.933, low-dose = 0.931, high-dose = 0.938) was worse than within genomic conditions, but was comparable to inter-genomic comparisons (WT vs. TRAM  =  0.926, WT vs. IRAK  =  0.918, TRAM vs. IRAK  =  0.942). This can also be seen in the representative H3K27ac tracks, where the pattern of the H3K27ac signal is similar across the WT and mutant cells, though also with some clear differences (Fig. 4b). To understand what effect that TRAM-deficiency has on WT enhancers, we analyzed the normalized H3K27ac signal of TRAM-deficient cells at the location of the WT enhancers, and compared the WT signal with the TRAM-deficient signal (Fig. 4c). We see that almost all of the enhancers have an opposite or differing pattern in TRAM-deficient cells than in WT cells, suggesting that TRAM-deficiency has an effect on enhancers in both chronically inflamed and exhausted monocytes. In Clusters I, II, and III, we see that TRAM plays a role in actin organization, leukocyte migration, and lymphocyte differentiation in both low-dose and high-dose, though often to different degrees. However, in Cluster X, we found that some of the enhancers associated with leukocyte migration and actin organization are uniquely increased in low-dose conditions in TRAM-deficient cells. In Cluster IV, we also see that TRAM is necessary for regulating leukocyte activation, which appears to be needed for low-dose conditions but not high-dose. This suggests that perhaps TRAM plays a role in preventing the overactivation of the immune system in low-grade inflammation.

We next performed a similar analysis of the effect of IRAK-M-deficiency on enhancers (Suppl. Fig. 3a). We did find noticeable differences with significant pathway enrichment in most of the clusters. In Cluster V, we see that the Myd88 pathway normally inhibits the transcription of primary miRNAs—this might be worth future investigation, as miRNAs play a role in regulating gene expression. In Cluster VIII, we see that, while the overarching pattern is the same, IRAK-deficiency shifts the low-dose profile closer to the high-dose profile, suggesting that IRAK-M has a small role in regulation here with regards to migration, reactive oxygen species metabolic processes, and endocytosis. In Cluster XI, we see that peptidyl-tyrosine phosphorylation is substantially reduced in IRAK-M-deficient low-dose and increased in high-dose. Tyrosine phosphorylation is a notable part of the LPS signaling cascade. It is possible that IRAK-M is necessary in low-grade inflammation for prolonged activation and in high-dose for inhibition. Cluster XII shows that IRAK-M deletion also appears to have an affect on enhancers associated with CD4+  T cell activation. One of the key genes in this cluster is *Tnfrsf1b*, which encodes *Tnfr2* and has been shown to promote survival in monocytes at low concentrations in a pathogenic environment^107^. Since the expression increases in low-dose IRAK-M-deficient cells, this suggests perhaps that the Myd88 pathway has a mild protective effect on monocytes in low-grade inflammation conditions. We also see that this cluster is related to Cluster VI. In both clusters, IRAK-deficiency leads to increased lymphocyte activation (or decrease negative regulation) in low-dose conditions, and to decreased activation in high-dose conditions. We also noticed that there seemed to be more consensus between H3K27ac signal of WT and IRAK-M-deficient cells at WT enhancer locations, and confirmed that TRAM-deficient cells primarily negatively correlated with WT cells, while IRAK-M-deficient cells positively correlated (Suppl. Fig. 3b). To determine if this was an artifact of only examining the enhancer signal at WT enhancer locations, we analyzed the distribution of signal at each of the respective, individual enhancer locations (i.e., WT, *Tram*−/−, *Irak-m*−/− etc.) and found that this trend holds in their respective enhancers as well (Suppl. Fig. 3c). While enhancer signal decreases with increasing LPS dose for WT and IRAK-M-deficient cells, enhancer signal increases with increasing LPS dose for TRAM-deficient cells. This suggests that the decrease in H3K27ac signal with increasing LPS dosage is possibly TRAM dependent.

### Transcriptomic changes due to low-dose and high-dose LPS exposure mirror epigenomic changes

**Fig. 5:**
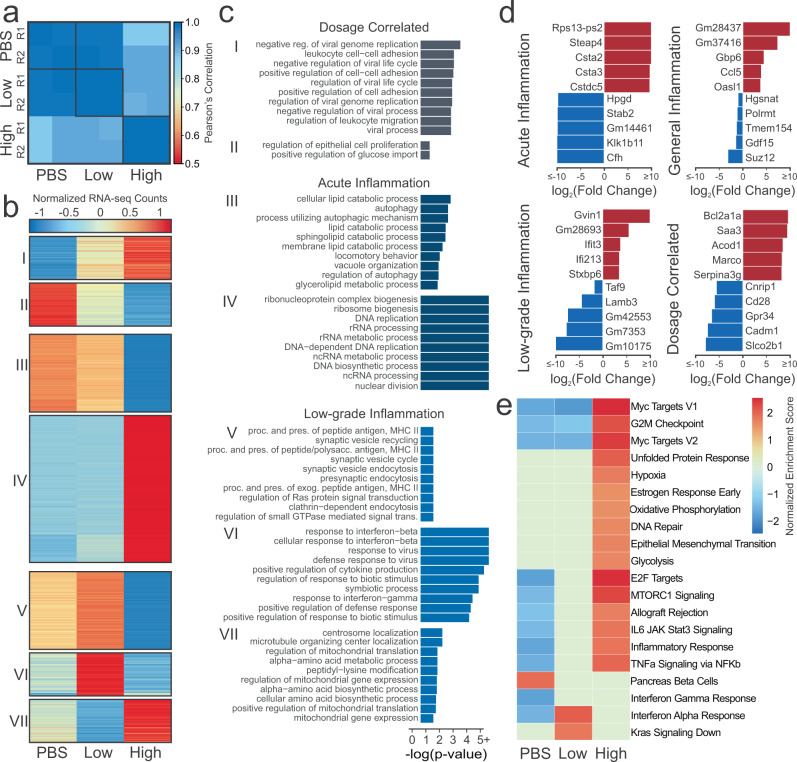
Effect of LPS dosage on gene expression. **a** Pearson’s correlation of normalized RNA-seq counts at genes **b** Heatmap of normalized gene expression of DEGs present in more than one comparison. **c** Significant biological process gene ontologies for genes that are differentially expressed in more than one comparison. **d** Top upregulated and downregulated genes for each condition. **e** Gene-set enrichment analysis. Color denotes normalized expression score if that pathway was significant (FDR < 0.05) in sample vs. rest.

We analyzed RNA-sequencing data to further understand the effect of LPS on murine immune cells. We see a similar pattern of RNA-seq data correlation as was in the ChIP-seq data (Fig. 5a). Average replicate correlation was 0.996 and the correlation between PBS and low-dose was similarly high (0.987). The high-dose replicates had approximately similar correlations with either PBS (0.892) or low-dose (0.914). Unlike the H3K27ac signal, there was not a decrease in RNA signal with increasing LPS-dosage (Suppl. Fig. 4a). Much like the differential peaks, the number of differentially expressed genes (DEGs) was lowest between PBS and low-dose (155) with much greater differences between PBS and high-dose (3,249) closely followed by low-dose and high-dose (3,061) (Suppl. Fig. 4b, Suppl. Data 4). However, differentially expressed genes were largely equally split into up- and down-regulated genes for each comparison, except between PBS and low-dose, where there were more genes upregulated in low-dose than PBS samples. Many genes (~63%) that are differentially expressed in one comparison, are differentially expressed in multiple comparisons (Suppl. Fig. 4c).

The normalized RNA-seq expression data of DEGs were visualized in a heatmap (Fig. 5b, Suppl. Data 5). Each of these clusters were analyzed for enrichment of Gene Ontology biological process gene sets (Fig. 5c, Suppl. Data 5). Dosage correlated Clusters I and II, which increased or decreased with added LPS-dosage, are consistent with enhancer analysis. Both analyses identified leukocyte-related pathways were increased with increasing dosage. In addition, negative viral process was also increased with additional LPS. While LPS is from a bacteria, TLR4 can also be activated by viral ligands, thus there is large overlap in the genes in each of these ontologies^108^. We also see an reduction of glucose import with increased LPS-dosage, which is also consistent with literature^109^. Clusters specific to acute inflammation, Clusters III and IV, had similar autophagy and nucleic acid-related processes as the corresponding enhancer clusters. However, Cluster III also had more lipid metabolic processes that were down-regulated in high-dose conditions, which is consistent with previous research showing LPS-stimulation to decrease lipid catabolism^110^.

Finally, Clusters V, VI, and VII were specific to low-dose. In Clusters V and VI, we see ontologies that are significantly upregulated in low-dose compared to high-dose or PBS. These include MHC II antigen processing and response to interferon-beta. MHC II antigen processing is the means by which an exogenous antigen is prepared and presented on the cell surface for the activation of CD4+  T cells. These cell surface molecules that present the antigen are only upregulated in the TRIF-dependent pathway. Furthermore, what the macrophage secretes can also affect CD4+  T cell function and aberrant CD4+  T cells have been implicated in inflammatory diseases^111,112^. Interferon-beta related genes and motifs were also found to be enriched or have increased enhancer expression in low-dose cells compared to high-dose or PBS. This suggests that epigenetics play a notable role in the etiology of low-grade inflammation. This is further supported by the peptidyl-lysine modification process in Cluster VII, which is down in low-dose and high in high-dose. With closer inspection, *Hdac2* and *Hdac4* are included within this clusters. *Hdac2* is considered to be crucial to the LPS inflammatory response while also mediating it, and decreased *Hdac2* levels have been found in COPD patients^113–115^. *Hdac4* is necessary for LPS-stimulated production of pro-inflammatory cytokines, and degradation leads to secretion of HMGB1, which is believed to play a substantial role in sepsis^116,117^.

The fold-changes of the top genes significantly expressed or repressed in exhaustive Acute Inflammation (high-dose vs. rest), Low-grade inflammation (low-dose vs. rest), General Inflammation (PBS vs. rest), and Dosage-specific (increasing or decreasing from PBS to high-dose) were inspected (Fig. 5d, Suppl. Data 5). In each, we see some genes that have been pointed out in literature before as affected by inflammation or LPS in some way, such as *Steap4*^118^, *Bcl2a1a*^119^, *Saa3*^120^, *Marco*^121^, *Cfh*^122^, *Ifit3*^123^, *Lrrc14b*^124^, *Ccl5*^125^, and *Cd28*^126^. We also see multiple predicted genes that might be worth further in vivo or in vitro functional investigation.

Gene-set enrichment analysis was then performed using each of the conditions compared to the rest (Fig. 5e). The first aspect of note is that high-dose is significantly enriching many pathways that have been found to be enriched by LPS-stimulation in literature such as Myc^127,128^, hypoxia^129^, glycolysis^130^, and unfolded protein response^131^. We also see oxidative phosphorylation, which is generally believed to be reduced in LPS-stimulation as the cell transfers to glycolysis, though there is some conflicting evidence^132^. Yet, there are two reasons that it might be enriched in high-dose. First, oxidative phosphorylation is necessary for inflammatory resolution^133^. Or, it could be as a result of glucose starvation, which can cause cells to shift back towards oxidative phosphorylation^134^. In addition, there are also pathways that seem to be somewhat dosage-dependent, such as E2F targets^135^, MTORC1^136^, IL6 JAK Stat3 signaling^137,138^, and TNF-α^139,140^ that are hallmarks of inflammation. However, it is interesting to note that it appears low-dose cells are not undergoing as much replication, as seen in the reduced G2M checkpoint and glycolysis. Furthermore, it appears that, while slight interferon-gamma enrichment^141,142^ occurs in both low-dose and high-dose (seen by deficiency in PBS), that low-dose has stronger enrichment of interferon-alpha and Kras signaling down. Interestingly, interferon-alpha overexpression, which studies suggest can lead to chronic inflammation^143^, is a characteristic of systemic lupus eruthematosus, an autoimmune disease^144,145^. The reduction in Kras signaling, which is part of the Ras/MAPK pathway, would also point to reductions in the cell-cycle of low-dose cells^146^.

As differential transcript usage (DTU), such as alternative splicing, has been shown to be affected in the macrophage inflammatory response^147^, we also chose to examine DTU among the three experimental conditions as differential transcript usage can effect the function of the genes involved. There were a total of 309 genes that had significant DTU with predicted functional consequences across the three comparisons (PBS vs. low-dose = 82, PBS vs. high-dose = 171, low-dose vs. high-dose = 172) (Suppl. Fig. 5a). Genes with DTUs in PBS versus High-dose were entirely associated with RNA-splicing ontologies (Suppl. Fig. 5b), as well as many in low-dose vs high-dose (Suppl. Fig. 5c). However, low-dose versus high-dose did have several immune related ontologies, suggesting that DTU may play a role in the differing responses. In particular, we see response to interferon-beta and one of the DTU genes that was involved, IFI204, plays a key role in interferon-beta release^148^. We also found that, of the 172 DTUs in low-dose vs. high-dose, 111 had (FDR  <  0.05) differential H3K27ac signal at their promoters (expected  =  40) and 38 were linked to enhancers (expected  =  22) for a total of 121 DTUs associated epigenetic regulation. This further suggests that epigenetic regulators may be playing a role in the differences between acute and low-grade inflammation.

### *Tram*−/− and *Irak-m*−/− each have roles under low-dose and high-dose LPS conditions

**Fig. 6:**
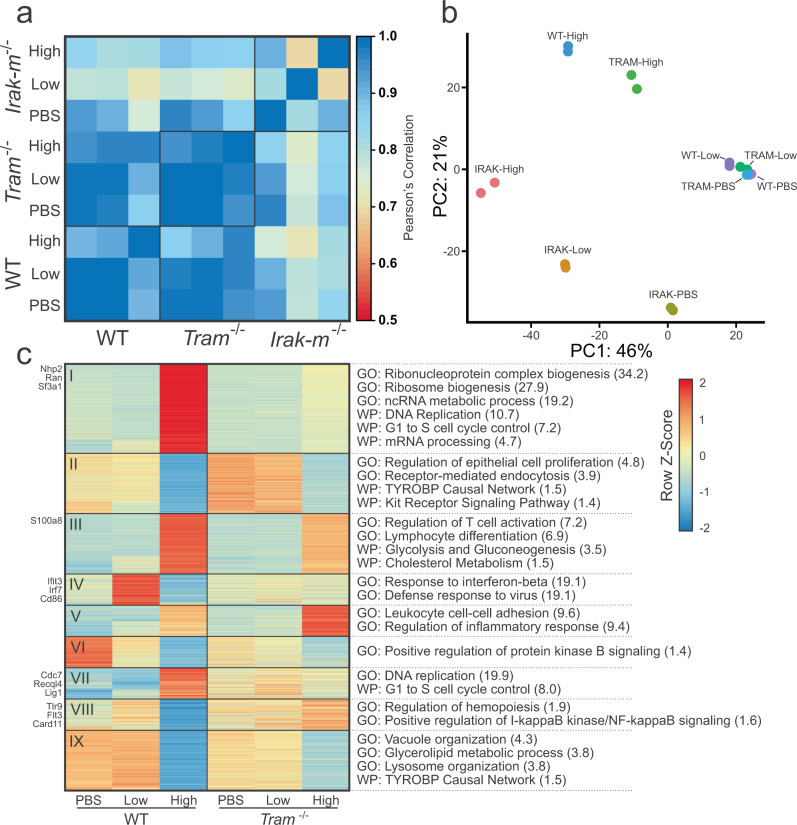
Effect of TLR4 pathway on LPS-dosage response. **a** Pearson’s correlation of RNA-seq signal between conditions. **b** Principle Component Analysis of replicates using top 500 variable genes. **c** Heatmap of WT and TRAM-deficient gene expression at varied LPS-dosage using DEGs from the WT comparisons. Significant pathways on right are from Gene Ontology or WikiPathways. Log(FDR) for each in parentheses.

The increase that we see of type I interferons-alpha and beta-related processes in low-dose cells, both epigenetically and transcriptomically, as well as increased epigenetic enhancement of TRAM (RNA-seq FDR  <  0.05, FC ≈ 1.8) led us to postulate that low-dose LPS might lead to more use of the TRIF-dependent pathway (which requires TRAM), while high-dose might lead to more use of the MyD88-dependent pathway (Fig. 3a and Fig. 5c, e)^37^. In order to better understand the mechanistic pathways that might be differentially affected by low-dosage of LPS, we performed additional experiments using TRAM-deficient and IRAK-M-deficient BMDMs (Fig. 6a).We see that WT-high-dose cells correlate the strongest with TRAM-deficient-high (*r*  =  0.961) followed by the low-dose and PBS conditions (*r*  =  0.866 – 0.915), while the IRAK-M samples had the lowest correlation (High  =  0.815, PBS  =  0.762). Average correlation between the WT and TRAM-deficient low-dose and PBS samples was very high at *r*  =  0.986. IRAK-M-deficient cells had the lowest average intra-group correlation (IRAK-M  =  0.803, TRAM  =  0.969, WT  =  0.934) and the lowest average inter-group correlation (IRAK-MvWT = 0.819, IRAK-MvTRAM = 0.847, WTvTRAM = 0.952). We can also see this pattern in the representative RNA-seq tracks of all three groups (Suppl. Fig. 6a). When we perform principle component analysis of the most variable genes, we see fairly similar results (Fig. 6b). These data were consistent with our initial independent flow-cytometry based studies, demonstrating that the expansion of low-grade inflammatory monocytes by low-dose LPS was completely TRAM dependent (Fig. 1), and that the exhaustion of monocytes induced by higher dose LPS was also partially TRAM dependent. In contrast, IRAK-M deficiency led to an expansion of the low-grade inflammatory monocyte when challenged with low-dose LPS, while IRAK-M deletion attenuated the magnitude of monocyte exhaustion in cells treated with high dose LPS. Due to the substantial differences in IRAK-M-deficient cells, we chose to focus more on the changes present in TRAM-deficient cells.

We then analyzed gene expression across the three dosing conditions for both WT and TRAM-deficient cells, though we restricted the analysis to genes that were differential expressed between the WT conditions (Fig. 6c, Suppl. Data 6). A decent number of genes were moderately affected by *Tram* deletion, but most maintained a similar expression pattern to WT. Despite this, there are 4 clusters of interest (Clusters I, IV, VII, and VIII) that stand out. Clusters I and VII both show a failure to upregulate DNA replication pathways in TRAM-deficient high-dose cells. While there is evidence of macrophage renewal due to inflammatory insult, it is not well understood, but it is possible that it is TRAM-dependent^149,150^. We also noticed that *S100a8* was preferentially induced by high dose LPS in WT cells, and less in TRAM deficient monocytes.

On the other hand, Clusters IV and VIII both have a pattern of low-dose having the highest expression and high-dose the lowest in WT. In Cluster VIII, *Tlr9* is an intracellular toll-like receptor that recognizes unmethylated CpG motifs in bacterial or viral DNA^151^. However, TLR9 activation has been shown to occur after LPS stimulation^152^ and TLR9 inhibition helps suppress excessive inflammation in bacterial sepsis^153–155^. Furthermore, it helps regulate antigen presentation in macrophages^156^ and participates in interferon-alpha production through IκB kinase signaling^157^. Interestingly, TLR9 is found to be activated through the NF-κB, ERK, JNK, and p38 MAPK pathways which are not TRIF-dependent, so it is unclear why there is not an increase in TRAM-deficient cells as well^152,158^.

Another critical gene is *Irf7*, which is upregulated in WT-low but not in TRAM-low, that is important for the production of type I interferons^159^. Furthermore, IRF7 expression can lead to a feed-forward loop of type I interferon production, much like we see in WT-low^160,161^. However, research has shown that IRF7 is necessary for the IL1B and the type I interferon response elicited through TLR4, and that IRF7 is induced through the TRIF-dependent pathway, consistent with the lack of IRF7 increase in TRAM-deficient cells. *Trim28* is a negative regulator of IRF7^159^ and is significantly reduced in WT-low compared to WT-high cells (FDR  <  0.05, FC  <  2), while remaining unchanged across the TRAM-deficient cells. If TRIM28 is phosphorylated at serine 473 through a PKR/p38 MAPK/MSK1 signaling cascade, it is no longer capable of inhibiting IRF7^162^. Although knockdown of activated *Msk1* does increase production of some inflammatory cytokines in neuroinflammation, TRIM28 is responsible for regulating a staggering number of genes, so it is unclear if it is involved^162,163^. In addition, phosphorylation of TRIM28 is not necessarily equivalent to reduced gene expression. FOXO3^164^ and CFLAR^165^ have also been shown to inhibit IRF7 but neither had notable differences in gene expression.

We performed a similar analysis of comparing the expression of WT DEG genes in WT and IRAK-M-deficient cells (Suppl. Fig. 6b). We see that Clusters I, II, IV, V, and VI had the most clear differences between the two cell-types, though Cluster III did not have any significantly enriched pathways. In Cluster IV, we see that there is much higher signal in low-dose IRAK-M-deficient cells, and that these genes are associated with a defense response to virus and response to interferon-beta. We previously saw that this was increased in low-dose WT cells (Fig. 5c), but it seems to be even further increased in IRAK-M-deficient low-dose, suggesting that the MyD88 pathway may have a role in regulating the low-dose response. Clusters V and VI generally show a loss of catabolic activity in PBS and low-dose conditions. Clusters II and VII show increases in metabolic pathways (shown as a decrease of negative regulation in Cluster VII) in high-dose conditions. In DNA replication, mTORC1 is responsible for inhibiting catabolic processes and possibly involved in purine and pyrimidine synthesis^166^. We note that WT high-dose already had increased mTORC1 signaling (Fig. 5e) and lower catabolic activity (Suppl. Fig. 6b). Therefore, this suggests that *Irak-m* deletion causes an increase in DNA replication activity across all conditions.

### TRAM is involved in the generation of exhausted monocytes

**Fig. 7:**
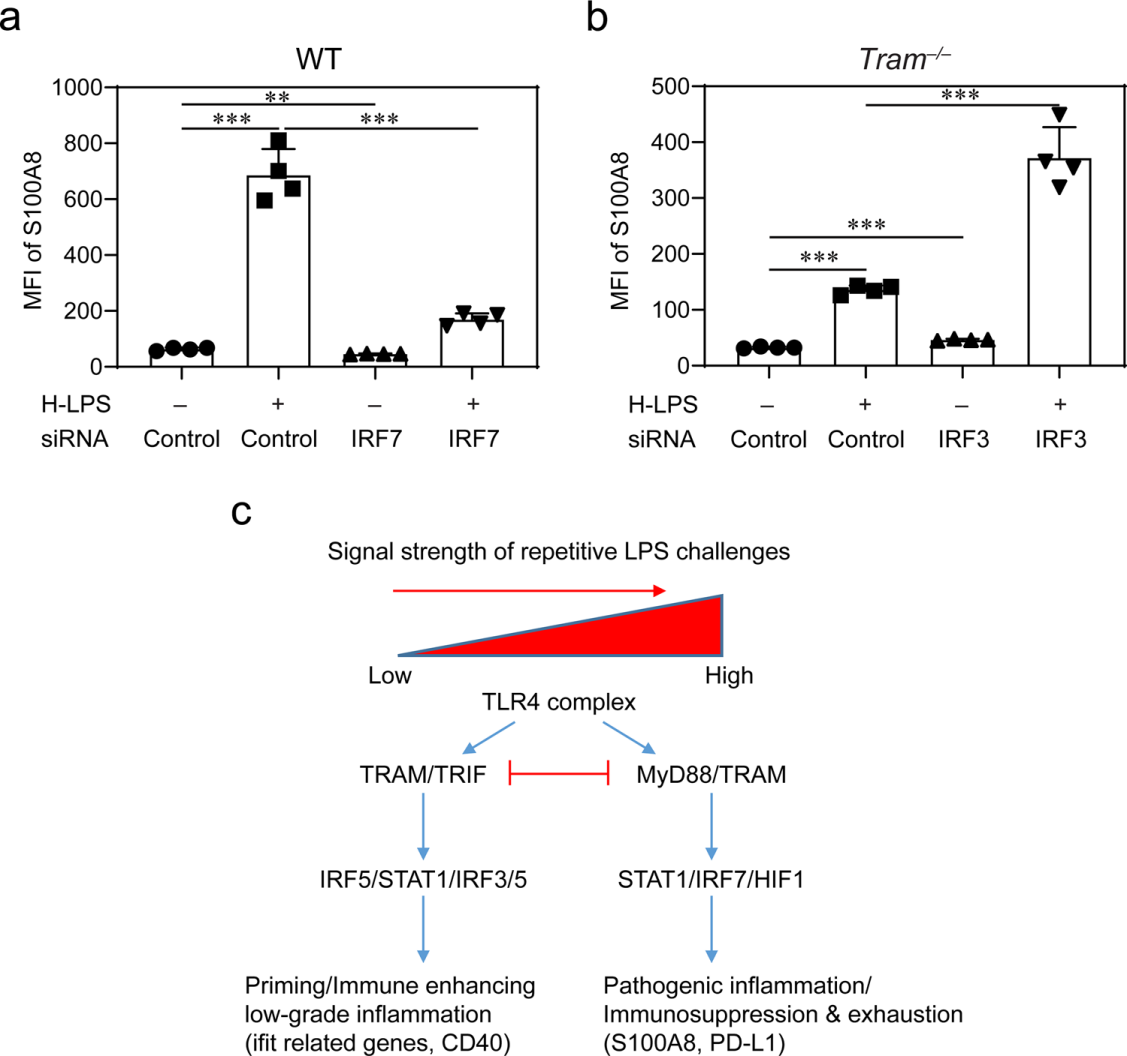
Causal contribution of TRAM-mediated IRF7 during the generation of exhausted monocytes. **a** WT monocytes were treated with high-dose LPS (1 µg/mL) in the presence of IRF7 siRNA or control siRNA for 5 days. The expression of S100A8 was determined with flow cytometry. **b** *Tram*^−/−^ monocytes were treated with high-dose LPS (1 µg/mL) in the presence of IRF3 siRNA or control siRNA for 5 days. The expression of S100A8 was determined with flow cytometry. Data are representative of three independent experiments, and error bars represent means ± SEM. **P < 0.01, and ***P < 0.001; one-way ANOVA (*n* = 4 for each group). **c** A schematic summary of the potential dynamics involved in the programming of inflammatory monocytes and exhausted monocytes.

Although the immune-enhancing effects of monocytes may be generated through TRIF^167^, it is not clear how monocyte exhaustion may develop. Our data suggest that with higher dose LPS challenge, TRAM, in contrast to TRIF, may distinctly mediate the generation of exhausted monocytes. Exhausted monocytes treated with high-dose LPS express pathogenic mediators such as S100A8, independently identified in monocytes from patients suffering from sepsis or COVID-19^168–170^. We observed that TRAM-deficient monocytes expressed less S100A8 following persistent challenges with high-dose LPS. We independently confirmed such findings with flow cytometry analyses (Fig. 7a, b, Suppl. Data 7). Based on the clue that IRF7 elevation is dependent upon TRAM, we further performed siRNA studies to confirm the causal connection between elevated IRF7 with the expression of S100A8^171^. As shown in Fig. 7, siRNA knock-down significantly suppressed the induction of S100A8 in WT monocytes challenged with high-dose LPS. IRF3 and IRF7 form a competitive circuit^171^, and reduction of IRF3 was shown to cause enhanced activation of IRF7 (Fig. 7). We then performed siRNA studies to knockdown IRF3 in TRAM-deficient monocytes. Figure 7 also shows that TRAM-deficient monocytes with *Irf3* knockdown robustly expressed S100A8, as compared to control TRAM-deficient monocytes.

Together, our data revealed that TRAM is uniquely involved in the generation of not only the inflammatory monocytes, but also the exhausted monocytes, likely through sustaining IRF7 activation (Fig. 7c).

## Discussion

Our genome-wide study of gene expression and histone modification changes suggest that low-grade inflammation condition (with persistent low-dose LPS challenges) leads to the generation of low-grade inflammatory monocytes with interferon signatures, while exhaustive inflammation (with persistent high-dose LPS stimulations) leads to a near complete depletion of non-classical monocytes and polarization of Ly6C++  monocytes, differentially modulated by TRAM. First, we found that these changes are seen in the variation of enhancers, the regulators of gene expression. Enhancers with increased signal in low-dose samples were linked to genes related to the TRAM/TRIF-dependent pathway. In addition, promoters and motifs enriched in low-dose samples were also associated with the interferon-beta response. Second, we showed that genes with increased expression preferentially in low-dose LPS, such as *Irgm1*, *Ifit3*, and *Bst2*, were significantly involved in interferon-beta and interferon-alpha responses. Furthermore, genes with differential transcript usage between low-dose and high-dose were also associated with interferon-beta response. Finally, we compared the gene expression of WT samples to TRAM-deficient mice to determine that TRAM-deficiency abrogates the interferon-beta associated genes, indicating that the low-dose increases in interferon-beta specifically rely on TLR4-associated TRAM/TRIF-dependent pathway, as opposed to MyD88 pathway. In the context of exhaustive inflammation, we observed that TRAM-mediated STAT1/IRF7 circuitry may be involved in the expansion of Ly6C++  monocytes with elevated induction of *S100a8*, a key signature gene elevated in septic patients including COVID-19 patients.

There is increasing appreciation for signal-strength dependent programming of both innate and adaptive immune systems, enabling complex and dynamic host responses to changing landscapes of infectious and inflammatory conditions^172,173^. Although much progress has been made with the dynamic programming of T helper cells exhibiting multi-staged activation and exhaustion under signal-strength and history dependent challenges^174^, the similar scenario of innate immune cell adaptation is still less understood. Due to limited systems approaches, even the most well-known concept of endotoxin tolerance regarding innate cell adaptation to repeated LPS challenges fails to clarify the complex innate immune adaptation dynamics. Past studies regarding endotoxin tolerance overly focused on dampened gene expression of limited inflammatory mediators^32^, and failed to address augmented induction of diverse immune-; metabolic-; and proliferative related genes involved in complex adaptation to higher dosages of endotoxin as increasingly recognized as the exhausted phenotype with dual features of pathogenic inflammation and immune suppression. Much less is known about innate responses to pathologically relevant subclinical low-dose LPS, highly prevalent in humans with chronic conditions due to mild leakage of mucosal barriers^175^. The lack of systems and clear understanding of signal strength and history-dependent adaptation to LPS underlies our limited translational success in treating related diseases ranging from acute sepsis to chronic cardiovascular diseases. Our current study provides a comprehensive assessment of gene expression dynamics as well as corresponding epigenetic variations in monocytes challenged with rising dosages of LPS.

Confirming limited previous studies, our collected data reveal that higher doses of LPS not only cause suppression of certain subsets of inflammatory genes, but also potently induce wide arrays of genes involved in altered immune metabolism and proliferative potential. Further characterization of these altered gene expression landscape may help better explain the compound phenotypes of pathogenic inflammation and immune-suppression observed in septic leukocytes collected from human sepsis patients and model murine septic animals^176,177^. In contrast, low-dose LPS preferentially induces inflammatory interferon responsive genes, recently shown to be expressed in inflammatory monocytes collected in vivo from various chronic inflammatory disease models including lupus and atherosclerosis^145,178,179^.

Our integrated analysis of epigenomic changes at enhancer and promoter regions complements our gene expression data, in further revealing the preferential usage of TRAM/TRIF pathway by low-dose LPS. Our finding is consistent with limited previous studies showing that the TRAM/TRIF-dependent pathway is favored in low-dose LPS conditions and critical for lesion development in atherosclerosis^33^. In addition, we identified signature transcription factors involved in monocyte activation by low-dose LPS such as IRF1, 5, and 7, with IRF5 previously reported to be involved in monocyte priming by low-dose LPS^59^. The preferential enrichment of additional transcription factors such as IRF2 and KLF4 is also interesting, and may provide additional insight regarding the regulation of low-grade inflammatory monocytes. Low-dose LPS also enhanced the expression of *Rab11a*, a molecule involved in endocytic recycling of TLR4, providing a further mechanistic explanation for our previous observation that internalization of TLR4-LPS complex and the activation of TRAM/TRIF pathway are required for sensing low-dose LPS^35^. It is worth to note that TRIF may play a beneficial role under septic conditions by preventing immune exhaustion and enhancing immune functions^180–182^. In contrast, our data suggest that TRAM may diverge with TRIF under the exhaustive condition with high dose LPS challenge, and facilitate monocyte exhaustion through elevated STAT1/IRF7 circuitry. Further studies are warranted to tease out the complex coordination and competition between MYD88, TRAM, TRIF and other related TLR4 adaptors in directing the adaption of monocytes in signal strength and history dependent fashion.

Collectively, our integrative systems study further clarifies the highly complex and dynamic adaptation of macrophages to rising dosages of LPS, and reveals more of the inner workings of underlying mechanisms, yet much more research will be required to fully understand how immune pathways and components interact in the dynamic ontogeny of macrophage activation states related to the etiology of low-grade inflammation. Studies that utilize time courses, additional LPS concentrations, and other transgenic mice all would be beneficial for truly unveiling complex dynamics of monocyte programming and memory including the relative stability and plasticity of reprogrammed cells both in vitro and in vivo. Although ample studies reveal the temporal and spatial dependent memory of adaptive immune cells such as T helper cells^183^, the extent of innate immune memory and its potential reversal still remains largely unknown. Information revealed in this report complements emerging studies that monocytes may adopt diverse states and/or intermediate states with overlapping features far exceeding the original simple paradigm of M1/M2 states^184^. Future extensive studies will be needed to address these important questions. Additional epigenomic studies may also reveal the causal relationships at play and possible therapeutic targets. Together, this could lead to identification of relevant molecular targets in human immune cells for future clinical applications.

## Methods

### Mice

C57/BL6 mice purchased from the Jackson Laboratory were bred and maintained in pathogenic-free conditions. Male 8–12-week-old mice were used in this study. The Institutional Animal Care and Use Committee (IACUC) approved all procedures performed on the mice. The TRAM-deficient and IRAK-M-deficient mice used were also on a C57/BL6 background.

### Cell culture

Crude BM cells were isolated from the mice and cultured as previously published^33^. Briefly, cells were pooled from 6 to 7 mice and split into three separate plates. The cells were cultured in RPMI complete media (RPMI 1640 with 10% FBS, 2 mM L-glutamine, and 1% penicillin/streptomycin) along with M-CSF (10 ng/mL) and either PBS, low-dose LPS (100 pg/mL), or high-dose LPS (1 µg/mL). Fresh LPS was added every two days and the cells were harvested after 5 days.

### Flow cytometry analyses

For analyzing subset proportions, the monocytes treated with LPS for 5 days were harvested and incubated with anti-CD16/-CD32 antibodies (1:100 dilution, BD Biosciences, no. 553141) to block Fc-receptors. The cells were stained with fluorochrome-conjugated anti-CD11b (1:200 dilution, BioLegend, no. 101226) and anti-Ly6C (1:200 dilution, BioLegend, no. 128006) antibodies, and PI (Sigma-Aldrich) was added before flow cytometry. For examining S100A8 expression, the monocytes were treated with high-dose LPS (1 µg/mL), and IRF7 siRNA (Thermo Fisher Scientific), IRF3 siRNA (Thermo Fisher Scientific), or control siRNA (Thermo Fisher Scientific) was mixed with Lipofectamine reagent to transfect monocyte cultures (25 pmol siRNA/well). Fresh LPS and siRNA were added every two days. After 5 days, the cells were fixed, permeabilized, stained with fluorochrome-conjugated anti-S100Ab antibody (1:100 dilution, Novus, no. NBP2-27067AF647), and then analyzed by flow cytometry.

### Chromatin shearing

Samples containing 10^6^ cells were centrifuged at 1600 g for 5 min at 4 °C. Each sample was washed twice with cold 1 mL PBS and resuspended in 9.375 mL of PBS. Then, 0.625 mL of 16% formaldehyde was added and the samples were incubated on a shaker for 5 min at room temperature. The samples were then quenched with 0.667 mL of 2 M glycine and incubated for 5 min at room temperature on a rotator. The cells were then centrifuged at 1600 g for 5 min and washed twice with 1 mL cold PBS. The pellet was resuspended in 130 µL of Covaris sonication buffer (10 mM Tris-HCl, pH 8.0, 1 mM EDTA, 0.1% SDS and 1× protease inhibitor cocktail (PIC)) and sonicated with a Covaris S220 sonicator using 75 W peak incident power, 5% duty factor, and 200 cycles per burst for 16 min at 4 °C. Sonicated samples were centrifuged at 16,100 × *g* for 10 min at 4 °C before the supernatant containing sheared chromatin was removed to a fresh tube. 2.4 µL of sheared chromatin was then mixed with 46.6 µL of IP buffer (20 mM Tris-HCl, pH 8.0, 140 mM NaCl, 1 mM EDTA, 0.5 mM EGTA, 0.1% (w/v) sodium deoxycholate, 0.1% SDS, 1% (v/v) Triton X-100, 1% freshly added PMSF and PIC) to generate a 50 µL sample containing chromatin from 20,000 cells for MOWChIP-seq.

### Bead preparation

IP buffer (20 mM Tris-HCl [pH 8], 140 mM NaCl, 1 mM EDTA, 0.5 mM EGTA, 0.1% (w/v) sodium doxycholate, 0.1% SDS, 1% (v/v) Triton-100X in Milli-Q water) was used to wash protein A-coated Dynabeads (Life Technologies). The beads were resuspended in 150 μL of IP buffer with 0.5 μg of H3K27ac (abcam, cat: ab4729, lot: GR312651-2) antibody, then rotated for 2 h at 4 °C. After rotation, the beads were washed with IP buffer three times before being resuspended in 5 μL of IP buffer and placed on ice.

### MOWChIP-seq

Sonicated chromatin samples of 20,000 cells per assay were profiled for H3K27ac (abcam, 0.5 µg per assay, cat: ab4729, lot: GR312651-2) with MOWChIP-seq as described in our previous publications^53,185^. Briefly, IP buffer is flowed into the device at a rate of 200 μL/min (30 psi) until all air is expelled. The sieve valve was closed and the antibody-coated beads were loaded into the device to form a packed bed. Then, the chromatin was flowed through at a rate of 1.5 μL/min. The beads were then washed in an oscillatory manner using first 50 μL of Low Salt washing buffer (20 mM Tris-HCl [pH 8], 150 mM NaCl, 2 mM EDTA, 0.1% SDS, 1% (v/v) Triton-100X in Milli-Q water) for five minutes at ~0.65 psi with the valve open and then another five-minute with High Salt washing buffer (20 mM Tris-HCl [pH 8], 500 mM NaCl, 2 mM EDTA, 0.1% SDS, 1% (v/v) Triton-100× in Milli-Q water). IP buffer was used to flow out the beads. Finally, the ChIP DNA was isolated from the beads by phenol-chloroform extraction. Libraries for sequencing were prepared using the Accel-NGS 2S Plus DNA Library Kit (Swift-Bio) and samples were sequenced using an Illumina HiSeq 4000 with single-end 50 nt reads.

### RNA-seq

In total 10,000 cells were used to produce each RNA-seq library, with two replicates for each genotype and experimental condition. RNA was extracted into a 30-µL volume using the RNeasy Mini Kit (74104, Qiagen) and RNase-Free DNase Set (79254, Qiagen), following the manufacturer’s instruction. The extracted mRNA was then concentrated by ethanol precipitation and resuspended in 4.6 μL of RNase-free water. Next, we used the SMART-seq2 protocol^55^, with minor modifications, to prepare cDNA. 2 μL of oligo-dT primer (100 μM) and 2 μL of dNTP mix (10 mM) were added to 2 ng of mRNA in 4.6 µL of water. The mRNA solution was denatured at 72 °C for 3 min, then immediately placed on ice. Next, 11.4 μL of reverse transcription mix [1 μL of SuperScript II reverse transcriptase (200 U/μL), 0.5 μL of RNAse inhibitor (40 U/μL), 4 μL of Superscript II first-strand buffer, 1 μL of DTT (100 mM), 4 μL of 5 M Betaine, 0.12 μL of 1 M MgCl_2_, 0.2 μL of TSO (100 μM), 0.58 μL of nuclease-free water] was added to the mRNA solution. For the reverse transcription reaction, the solution was incubated at 42 °C for 90 min, followed by 10 cycles of 50 °C for 2 min, 42 °C for 2 min, then inactivation at 70 °C for 15 min. 20 μL of the resulting solution (first-strand mixture) was then mixed with 30 μL of PCR mix [25 μL KAPA HiFi HotStart ReadyMix, 0.5 μL IS PCR primers (10 μM), 0.5 μL Evagreen dye, and 4 μL nuclease-free water] and amplified using the program 98 °C for 1 min, followed by 9–11 cycles of 98 °C 15 s, 67 °C 30 s, 72 °C 6 min. Finally, the cDNA was purified using 50 μL of SPRIselect beads. RNA-seq libraries were generated with the Nextera XT DNA Library Preparation kit (FC-131-1024, Illumina) and manufacturer’s protocol, using approximately 600 pg of purified cDNA from each sample. Samples were sequenced using an Illumina HiSeq 4000 with single-end 50 nt reads.

### Data processing

Unless otherwise mentioned, all data analysis was performed with Bash scripts or with R (The R Foundation) scripts in RStudio. Sequencing reads were trimmed using default settings by Trim Galore! (Babraham Institute). Trimmed reads were aligned to the mm10 genome with Bowtie^186^. Peaks were called using MACS2 (*q*  <  0.05)^187^. Blacklisted regions in mm10 as defined by ENCODE were removed to improve data quality^188^. Mapped reads from ChIP and input samples were extended by 100 bp on either side (250bp total) and a normalized signal was calculated using Eq. (1).

1 $${Normalized}\,{Signal}=\left(\frac{{ChIP}\,{Signal}}{{No}.\,{of}\,{ChIP}\,{Reads}}-\frac{{Input}\,{Signal}}{{No}.\,{of}\,{Input}\,{Reads}}\right)\times {10}^{6}$$

For Pearson’s correlation, the signal was calculated around the promoter region (TSS ± 2 kb) and plotted with the corr and levelplot functions. For visualization in IGV (Broad Institute), the signal was calculated in 100bp windows over the entire genome and output as a bigWig file. RNA-seq data was quantified using Salmon^189^ against the mm10 transcriptome using a full decoy and normalized counts were calculated with DESeq2^190^.

### Enhancers analysis

To call enhancers, we considered H3K27ac^high^ regions that did not intersect with promoter regions to be enhancer regions. First, consensus H3K27ac peak sets were generated for each of the experimental conditions. Peak widths were expanded to be 1000 bp long (summit ± 500 bp). Promoters were defined as TSS ± 2000 bp. Any H3K27ac 1 kb regions that intersected with a promoter region was removed and the remaining regions were designated as putative enhancers. The signal at each of the putative enhancers was then correlated (Spearman) to RNA-seq gene expression values within the same topological domain. Putative enhancers were linked to the gene with the highest correlation, however, links were only considered significant if the Spearman correlation coefficient (SCC) > 0.25 and if the correlation was considered significant if both empirical and quantitative *p*-values were less than 0.05. For both *p*-values, the SCC was calculated between the given putative enhancer and all genes on the same chromosome. The empirical *p*-value was then calculated as the fraction of genes on the same chromosome that has a higher correlation than the currently linked gene. The quantitative *p*-value was calculated by treating the calculated SCC values as a distribution and using the R function pnorm to calculate a significance. Enhancers were quantified using DiffBind^191^. Motif analysis was performed to determine enriched transcription factor binding motifs among the enhancer regions with HOMER^192^ (with options –size 1000 –mask –p 16 –nomotif).

### RNA-seq analysis

Differential gene expression analysis was performed using DESeq2^190^, where genes with a fold-change > = 2 and FDR < 0.05 were considered to be significantly differentially expressed. Boxplots and MA plots were done in R using ggplot and ggpubr. Clustering was performed using clusterProfiler. Gene-set enrichment analysis was performed with GSEA^193,194^ using the Hallmark gene set and gene-set level permutation. Gene sets were considered significant if the FDR < 0.05. Significant differential transcript usage (*p* < 0.05, dIF > 0.1) was determined using IsoformSwitchAnalyzeR^195,196^ with the default DEXSeq^197^. Data output was then ran through CPAT^198^, PFAM^199^, SignalP^200^, NetSurfP-2.0^201^, and results were combined back into IsoformSwitchAnalyzeR to determine genes that might have functional consequences as a result of the DTUs. Genes were analyzed for gene ontologies with clusterProfiler.

### Statistics and reproducibility

For each genotype (i.e. WT, *Tram*−/−, *Irak-M*−/−), cells from at least *n* = 6 mice were pooled and split into three experimental groups. Two technical replicates were analyzed from each genomic/experimental combination. Differences in genomic signal were calculated using unpaired t-tests and corrected for multiple testing using the Benjamini-Hochberg procedure. False discovery rates (FDR) were significant if FDR < 0.05. Unless specified otherwise, p-values were considered significant if *p* < 0.05. Flow cytometry was performed on *n* = 4 mice for each genotype and analyzed with a one-way ANOVA.

### Reporting summary

Further information on research design is available in the Nature Research Reporting Summary linked to this article.

## Supplementary information

Supplementary Information

Description of Additional Supplementary Files

Supplementary Data 1

Supplementary Data 2

Supplementary Data 3

Supplementary Data 4

Supplementary Data 5

Supplementary Data 6

Supplementary Data 7

Reporting Summary

## Data Availability

The ChIP-seq and RNA-seq data sets are deposited in the Gene Expression Omnibus (GEO) repository with the following accession number: GSE168190. All other data is available from the corresponding author on reasonable request. Source values for each of the following figures are available in the corresponding Supplementary Data files: Fig. 1 – Supplementary Data 1; Fig. 2 – Supplementary Data 2; Fig. 3 – Supplementary Data 3; Supplementary Fig. 4 – Supplementary Data 4; Fig. 5 – Supplementary Data 5; Fig. 6 – Supplementary Data 6; Fig. 7 – Supplementary Data 7.

## Code availability

We used the following software in the analysis**:** R v3.6.1 (The R Foundation), Trim Galore! v.0.4.1 (Babraham Institute), Bowtie v1.1.2, MACS2 v2.1.1.20160309 (*q* < 0.05), IGV v2.4.10 (Broad Institute), DiffBind v2.12.0, Salmon v1.2.1, DESeq2 v1.30.0, HOMER v4.10.3 (with options –size 1000 –mask –p 16 –nomotif), clusterProfiler v3.18.0, GSEA 4.1.0, IsoformSwitchAnalyzeR v3.12 (*p* < 0.05, dIF > 0.1), DEXSeq v1.35.1, CPAT v3.0.2, PFAM (HMMER) v2.41.1, SignalP v5.0, NetSurfP-2.0. All codes can be made available upon reasonable request.
