# Supplementary material for: Epigenomic and transcriptomic analyses reveal differences between low-grade inflammation and severe exhaustion in LPS-challenged murine monocytes
Source: Commun Biol. 2022 Jan 28;5:102. doi: 10.1038/s42003-022-03035-2 (PMC8799722; doi:10.1038/s42003-022-03035-2)
Supplement: Supplementary file 1 — Supplementary Information [file 42003_2022_3035_MOESM1_ESM.pdf]

Supplementary Figures

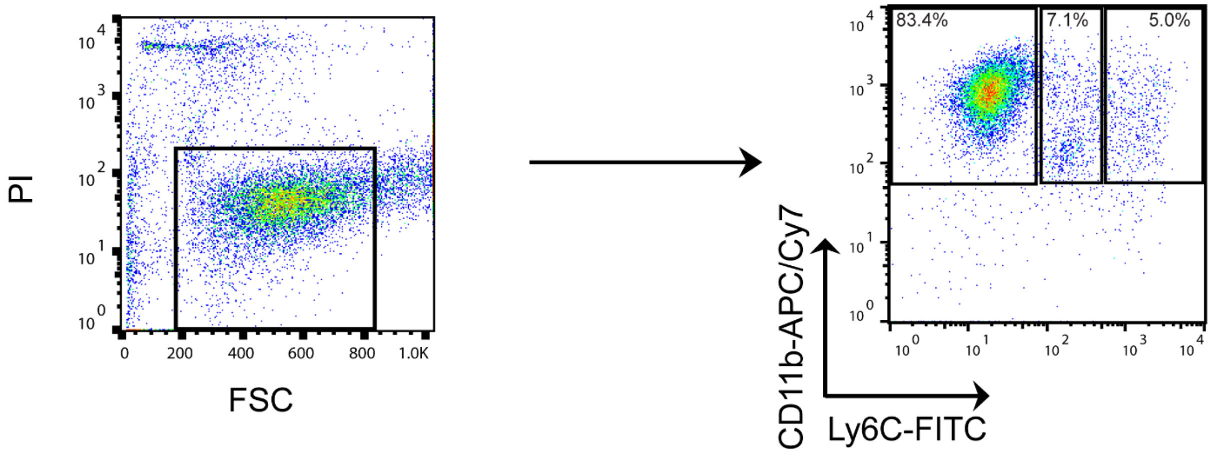

Supplementary Figure 1 Flow gating strategy for isolation of murine monocytes.

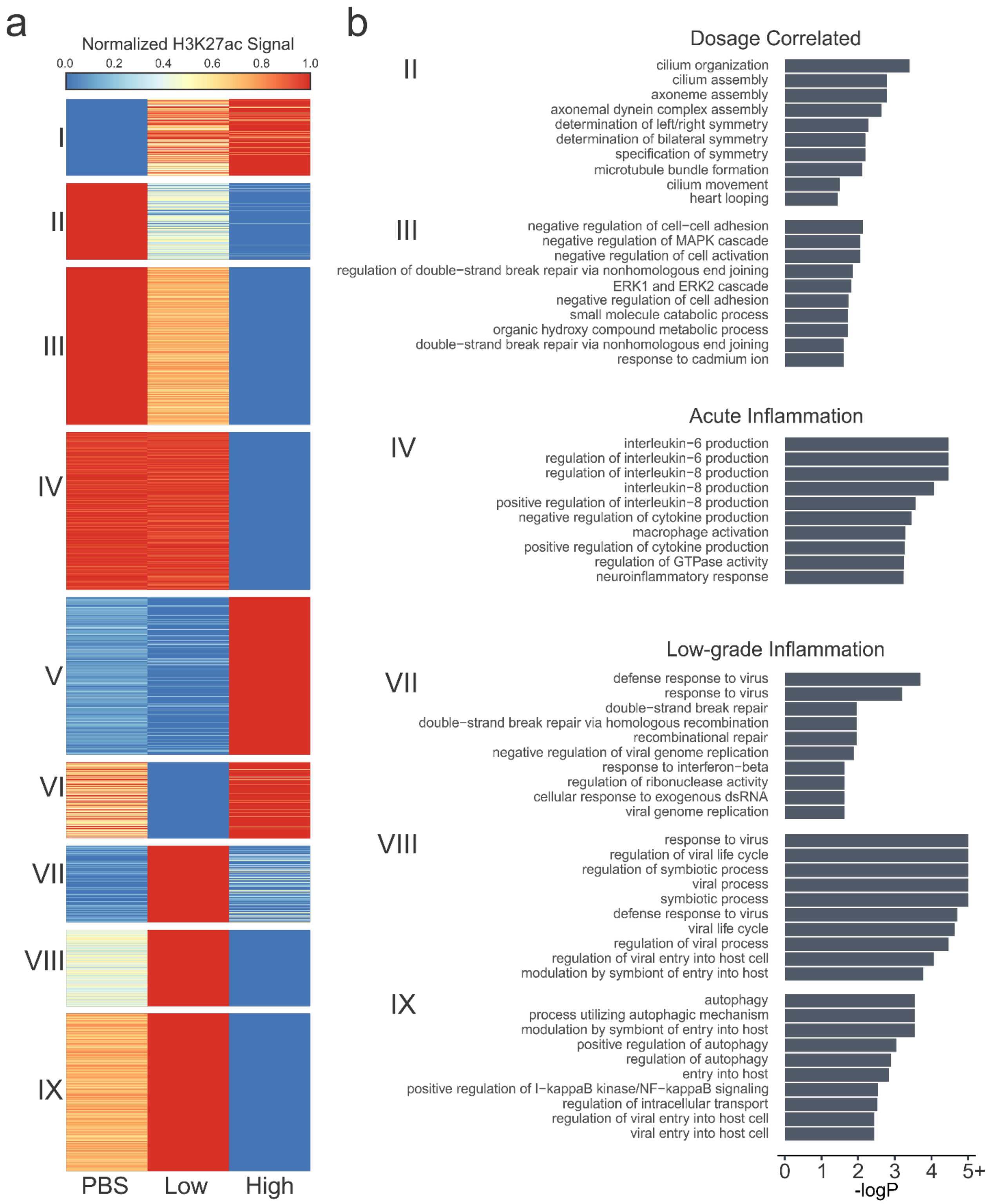

Supplementary Figure 2 H3K27ac signal at promoters for murine BMDMs dosed with PBS, low-dose LPS, or high-dose LPS. (a) Normalized H3K27ac signal around promoter regions (TSS +/- 2 kb) with differential marking (FDR < 0.05, fold-change >= 2) clustered using k-means clustering. (b) Gene ontologies that are significantly enriched (FDR < 0.05) in the associated clusters from (a).

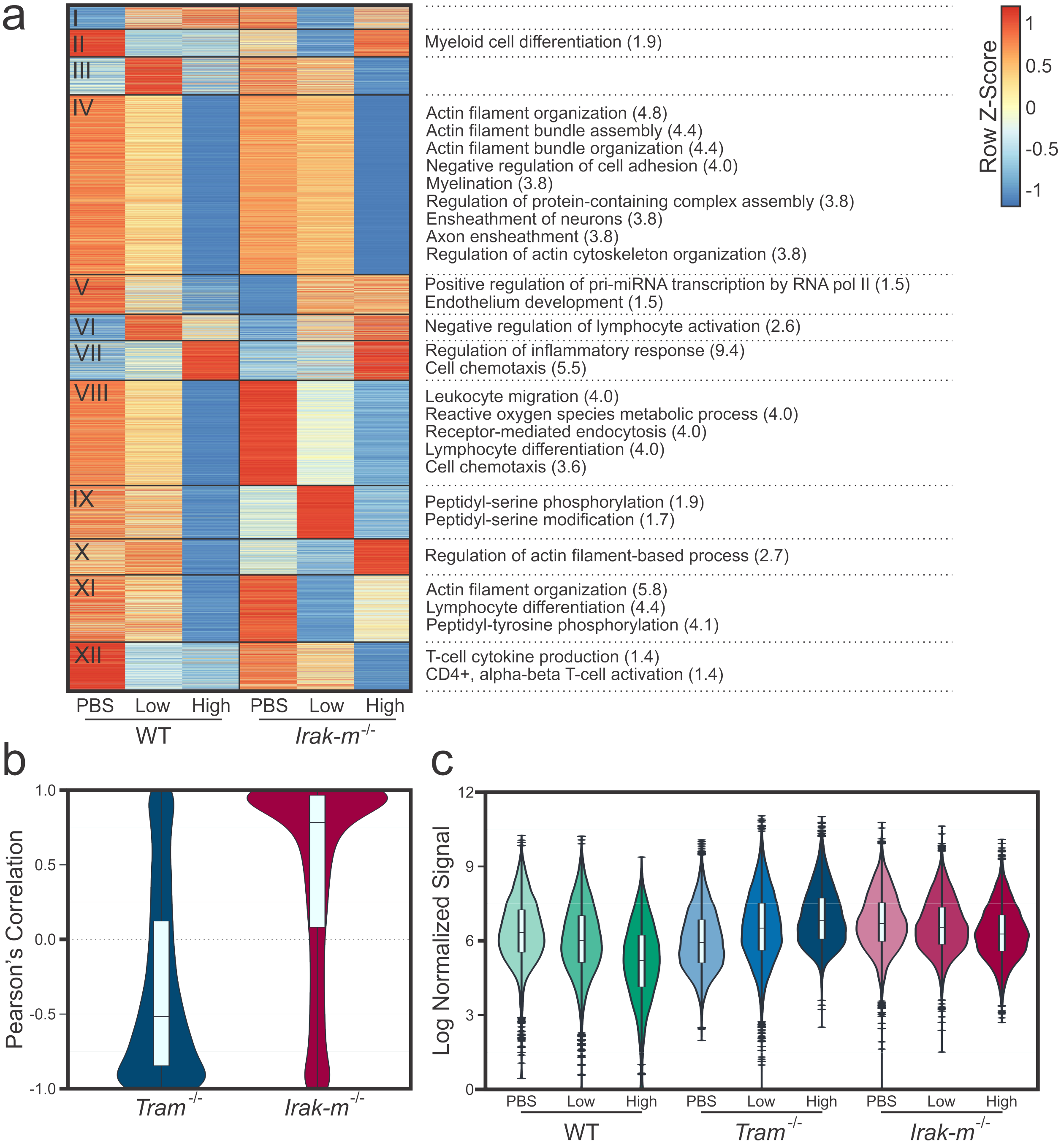

Supplementary Figure 3 Effect of mutation on WT enhancers (a) Normalized H3K27ac signal at WT enhancers in WT and IRAK-deficient cells. Significant biological process gene ontologies (FDR < 0.05) listed on the right, with log(FDR) in parentheses. (b) Pearson's correlation of normalized H3K27ac signal of TRAM-deficient cells or IRAK-M-deficient cells with WT cells at WT enhancer locations (*n* = 6,737 enhancers). (c) Normalized H3K27ac signal of enhancers specific to each genotype (WT *n* = 6,737; *Tram-/- n* = 10,743; *Irak-m-/- n* = 6,179). The middle bar of the boxplot denotes the median. The upper and lower bounds of the box correspond to the 75th and 25th percentile, respectively. Each whisker spans up to 1.5 * interquartile range from the edge of the box.

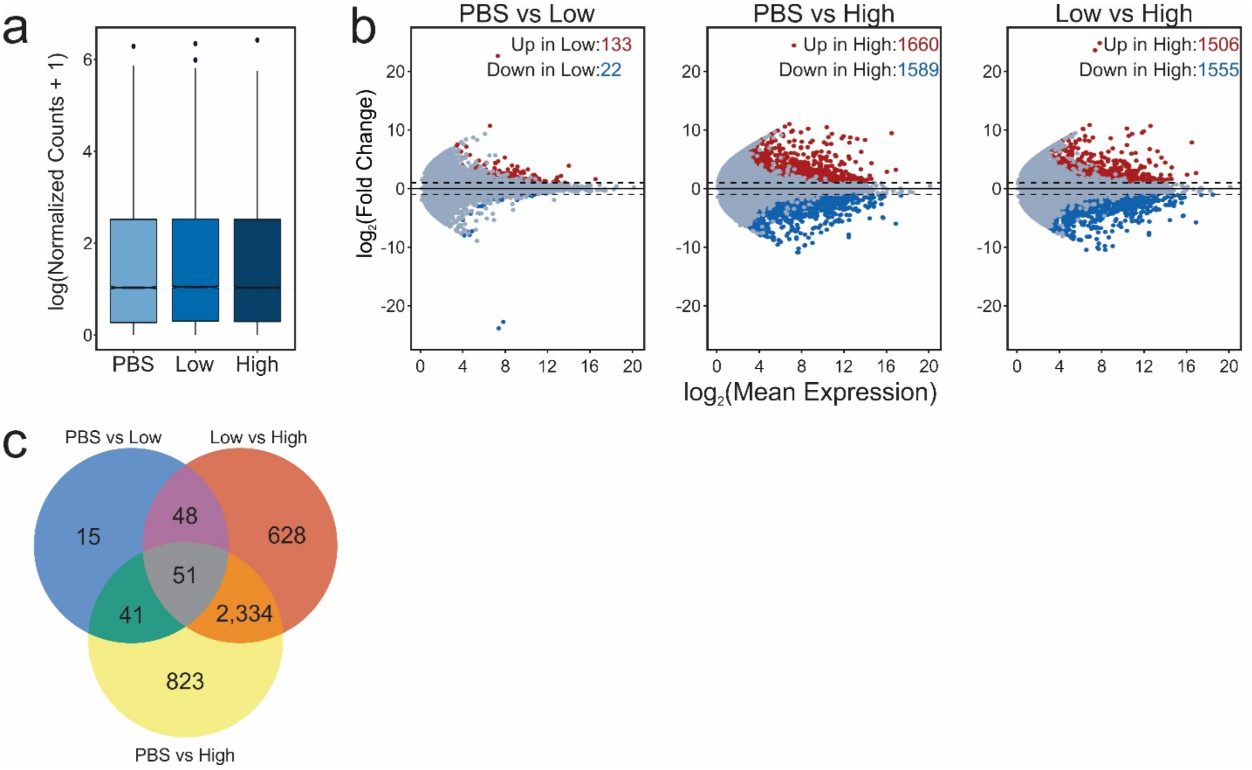

Supplementary Figure 4 Overview of gene expression changes due to LPS. (a) Boxplots of normalized RNA-seq counts at genes (*n* = 54,347). The middle bar of the boxplot denotes the median. The upper and lower bounds of the box correspond to the 75th and 25th percentile, respectively. Each whisker spans up to the range of 1.5 * interquartile range from the edge of the box. (b) MA-plots of the fold change between two samples of a comparison versus the mean gene expression of that gene. Color denotes significance (FDR < 0.05, FC >= 2). (c) Overlap of differentially expressed genes.

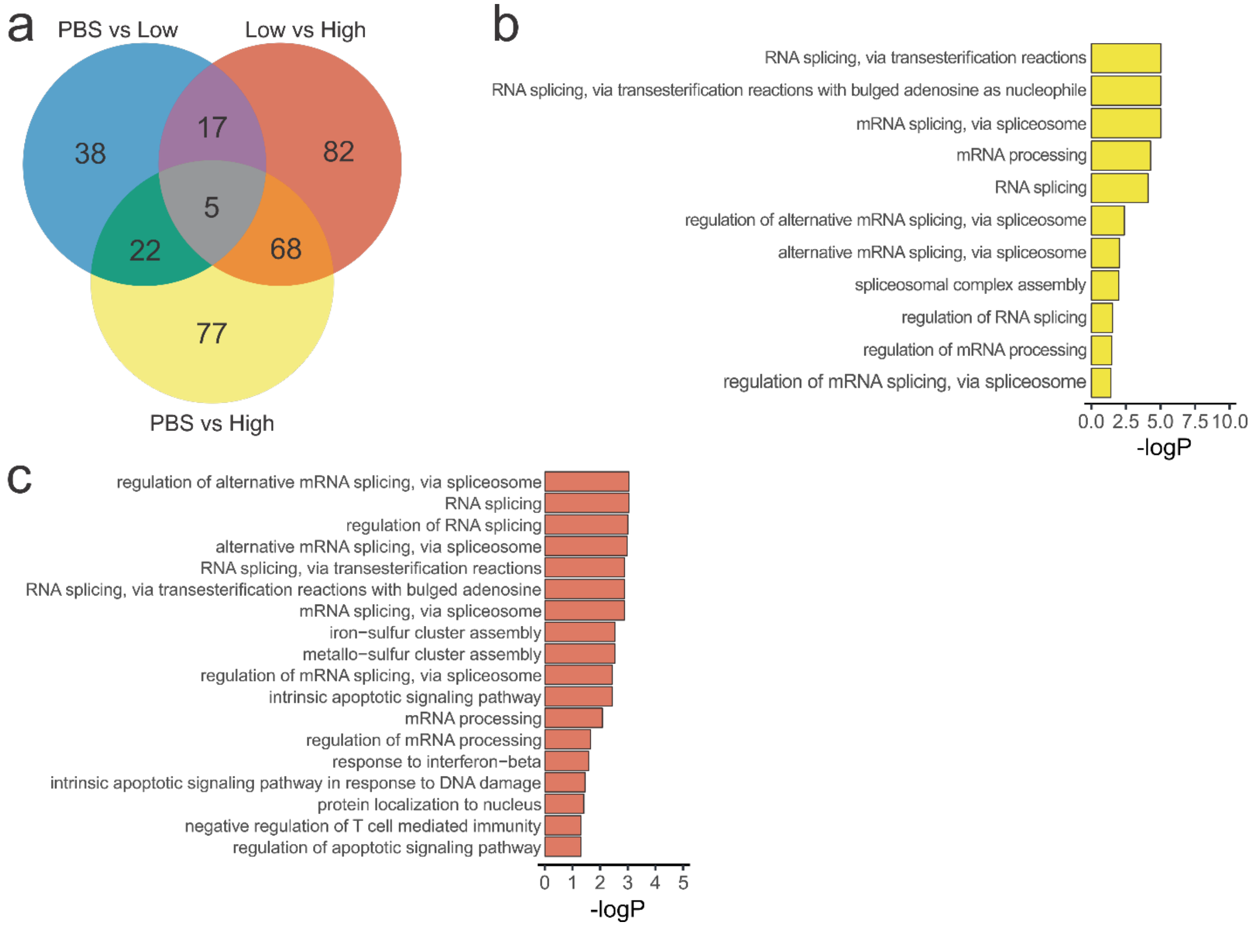

Supplementary Figure 5 Effect of LPS on differential transcript usage (a) Overlap of genes with significant differential transcript usage among the three conditions. (b) Gene ontologies significantly overrepresented in genes with DTUs in High-dose compared to PBS. (c) Gene ontologies significantly overrepresented in genes with DTUs in High-dose compared to Low-dose.

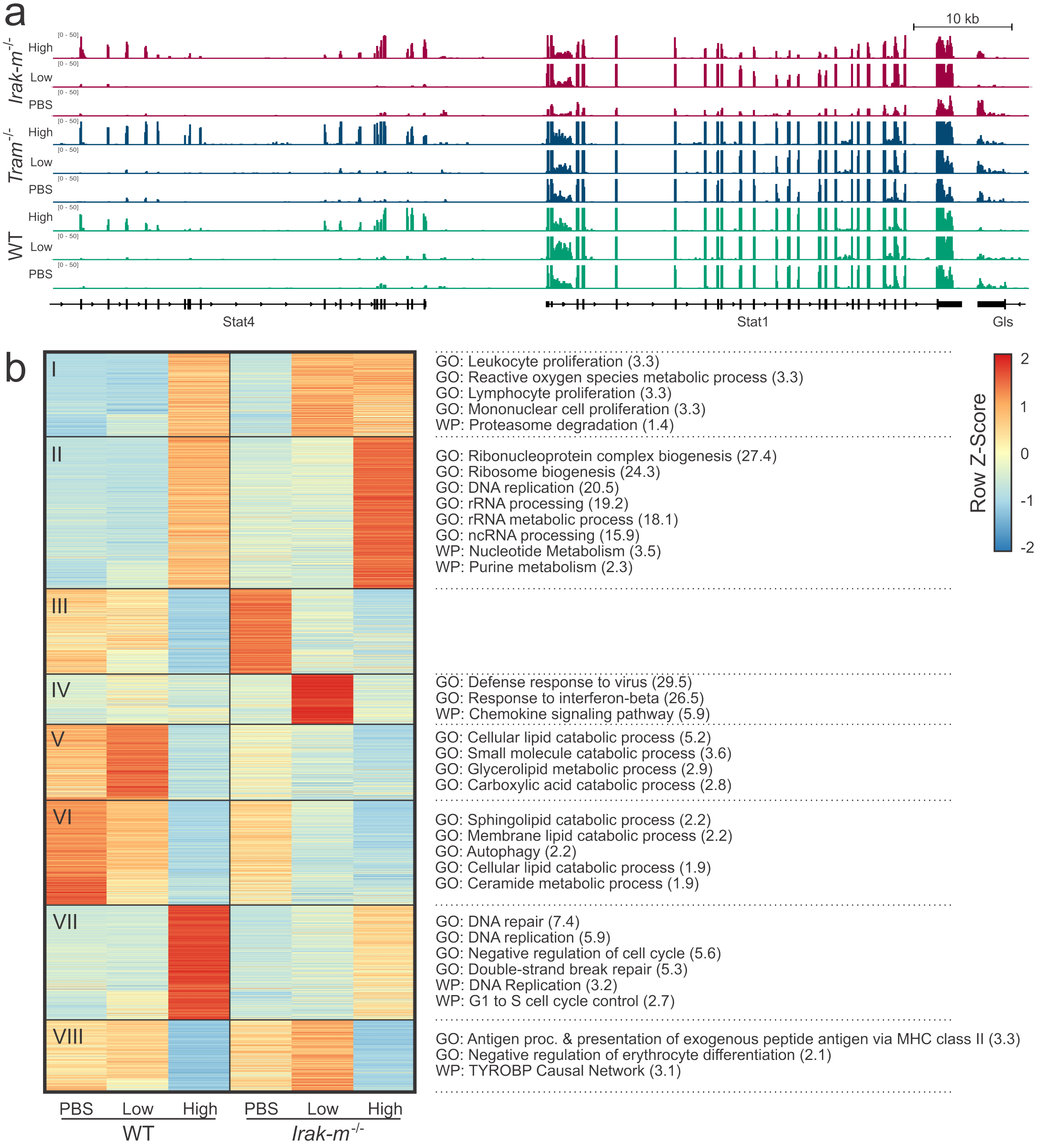

Supplementary Figure 6 Effect of IRAK-M deficiency on LPS-dosage response (a) Representative RNA-seq tracks of WT, TRAM-deficient, and IRAK-M-deficient cells. Tracks are aligned in mm10 and the region displayed is chr1:52,069,008-52,169,007. (b) Heatmap of WT and IRAK-M-deficient gene expression at varied LPS-dosage using DEGs from the WT comparisons. Significant pathways on right are from Gene Ontology or WikiPathways. Log(FDR) for each in parentheses.

Supplementary Tables

Table 1: H3K27ac ChIP-seq Metadata

	Sample	Total Reads (millions)	Trimmed Reads (millions)	Aligned Reads (millions)	Alignment (%)	Peaks	Consensus Peaks
WT	PBS-1	13.8	13.8	12.0	87.1	34,372	25,679
	PBS-2	15.9	15.9	14.0	88.0	46,067	
	Low-1	21.4	21.3	18.2	85.4	34,967	21,597
	Low-2	19.4	19.4	16.8	86.8	28,091	
	High-1	19.4	19.4	16.6	83.5	20,685	14,659
	High-2	21.0	21.0	18.1	86.2	23,775	
Tram$^{-/-}$	PBS-1	36.1	35.8	25.9	72.4	22,255	18,256
	PBS-2	34.4	34.0	23.5	69.2	29,432	
	Low-1	15.6	15.3	10.5	68.2	34,552	23,353
	Low-2	25.3	24.8	17.9	72.0	28,274	
	High-1	20.2	20.1	13.7	68.2	41,880	32,368
	High-2	21.8	21.5	14.6	67.9	45,166	
Irak-m$^{-/-}$	PBS-1	17.8	17.1	11.1	64.6	22,651	18,256
	PBS-2	22.7	21.9	13.8	63.0	34,861	
	Low-1	16.9	16.5	11.2	67.7	28,487	23,353
	Low-2	37.5	36.2	25.0	69.0	27,880	
	High-1	48.4	48.0	33.2	69.2	38,542	32,368
	High-2	28.1	27.9	19.7	70.5	20,557	

Table 2: RNA-seq Metadata

Genotype	Sample	Total Reads (millions)	Trimmed Reads (millions)	Aligned Reads (millions)	Alignment (%)
WT	PBS-1	18.0	18.0	15.0	83.4
	PBS-2	16.9	16.9	14.3	84.7
	Low-1	15.6	15.6	12.9	83.2
	Low-2	15.1	15.1	12.6	83.8
	High-1	16.8	16.8	14.2	84.5
	High-2	14.0	14.0	11.9	85.3
Tram$^{-/-}$	PBS-1	13.7	13.7	10.9	79.5
	PBS-2	14.2	14.2	11.7	82.2
	Low-1	14.0	14.0	11.3	80.7
	Low-2	15.0	14.9	11.8	79.1
	High-1	30.4	30.4	25.2	83.1
	High-2	14.2	14.2	11.0	77.9
Irak-m$^{-/-}$	PBS-1	13.4	13.4	11.1	82.7
	PBS-2	17.7	17.7	14.8	83.8
	High-1	16.2	16.2	13.8	85.4
	High-2	18.3	18.3	14.7	80.7
