# Supplementary material for: Epigenomic and transcriptomic analyses reveal differences between low-grade inflammation and severe exhaustion in LPS-challenged murine monocytes
Source: Commun Biol. 2022 Jan 28;5:102. doi: 10.1038/s42003-022-03035-2 (PMC8799722; doi:10.1038/s42003-022-03035-2)
Supplement: Supplementary file 2 — Description of Additional Supplementary Files [file 42003_2022_3035_MOESM2_ESM.pdf]

Description of Additional Supplementary Files

Supplementary Data 1: Percentage of monocyte subpopulations in WT, *Tram*-/-, and *Irak-m*-/- in PBS, Low-dose and High-dose

Supplementary Data 2: Effect of LPS dosage on H3K27ac peak signal, peaks lost/gained, and peak localization

Supplementary Data 3: Clustered normalized H3K27ac at enhancers with linked genes and corresponding gene ontology

Supplementary Data 4: Differentially expressed genes between WT experimental conditions

Supplementary Data 5: Clustered RNA-seq counts of genes differentially expressed in WT and corresponding gene ontology

Supplementary Data 6: Clustered WT and TRAM$^{-/-}$ RNA-seq counts of genes differentially expressed in WT and corresponding gene ontology

Supplementary Data 7: Effect of IRF7 and IRF3 respectively on expression of S100A8 in WT and *Tram*-/-
