# Supplementary material for: Epigenomic and transcriptomic analyses reveal differences between low-grade inflammation and severe exhaustion in LPS-challenged murine monocytes
Source: Commun Biol. 2022 Jan 28;5:102. doi: 10.1038/s42003-022-03035-2 (PMC8799722; doi:10.1038/s42003-022-03035-2)
Supplement: Supplementary file 10 — Reporting Summary [file 42003_2022_3035_MOESM10_ESM.pdf]

nature research

Corresponding author(s): Chang Lu

Last updated by author(s): Dec 29, 2021

Reporting Summary

Nature Research wishes to improve the reproducibility of the work that we publish. This form provides structure for consistency and transparency in reporting. For further information on Nature Research policies, see our Editorial Policies and the Editorial Policy Checklist.

Statistics

For all statistical analyses, confirm that the following items are present in the figure legend, table legend, main text, or Methods section.

n/a	Confirmed	
☐	☒	The exact sample size (*n*) for each experimental group/condition, given as a discrete number and unit of measurement
☐	☒	A statement on whether measurements were taken from distinct samples or whether the same sample was measured repeatedly
☐	☒	The statistical test(s) used AND whether they are one- or two-sided<br>*Only common tests should be described solely by name; describe more complex techniques in the Methods section.*
☒	☐	A description of all covariates tested
☐	☒	A description of any assumptions or corrections, such as tests of normality and adjustment for multiple comparisons
☒	☐	A full description of the statistical parameters including central tendency (e.g. means) or other basic estimates (e.g. regression coefficient) AND variation (e.g. standard deviation) or associated estimates of uncertainty (e.g. confidence intervals)
☒	☐	For null hypothesis testing, the test statistic (e.g. *F*, *t*, *r*) with confidence intervals, effect sizes, degrees of freedom and *P* value noted<br>*Give P values as exact values whenever suitable.*
☒	☐	For Bayesian analysis, information on the choice of priors and Markov chain Monte Carlo settings
☒	☐	For hierarchical and complex designs, identification of the appropriate level for tests and full reporting of outcomes
☒	☐	Estimates of effect sizes (e.g. Cohen's *d*, Pearson's *r*), indicating how they were calculated

Our web collection on statistics for biologists contains articles on many of the points above.

Software and code

Policy information about availability of computer code

Data collection: No software was used in data collection

Data analysis:
Trim Galore!: 0.4.1
fetchChromSizes: 332
bowtie: 1.1.2
bedtools: 2.26.0
samtools: 1.9
bedGraphToBigWig : 332
bedSort: 332
MACS2: 2.1.1.20160309
cor (stats): 3.6.0
ggplot2: 3.3.1
HOMER: 4.10.3
clusterProfiler: 3.18.0
Salmon: 1.2.1
IsoformSwitchAnalyzeR: 3.12
CPAT
SignalP: 5.0
Pfam: 2.41.1
NetSurfP2.0

For manuscripts utilizing custom algorithms or software that are central to the research but not yet described in published literature, software must be made available to editors and reviewers. We strongly encourage code deposition in a community repository (e.g. GitHub). See the Nature Research guidelines for submitting code & software for further information.

Data

Policy information about availability of data

All manuscripts must include a data availability statement. This statement should provide the following information, where applicable:

- Accession codes, unique identifiers, or web links for publicly available datasets
- A list of figures that have associated raw data
- A description of any restrictions on data availability

The ChIP-seq and RNA-seq data sets are deposited in the Gene Expression Omnibus (GEO) repository with the following accession number: GSE168190
https://www.ncbi.nlm.nih.gov/geo/query/acc.cgi?acc=GSE168190

Field-specific reporting

Please select the one below that is the best fit for your research. If you are not sure, read the appropriate sections before making your selection.

☒ Life sciences ☐ Behavioural & social sciences ☐ Ecological, evolutionary & environmental sciences

For a reference copy of the document with all sections, see nature.com/documents/nr-reporting-summary-flat.pdf

Life sciences study design

All studies must disclose on these points even when the disclosure is negative.

Sample size	We estimated sample sizes on the basis of our previous experience with epigenomic and transcriptomic assays on mouse samples.
Data exclusions	For WT ChIP-seq samples, only the two replicates with the highest number of peaks for each PBS, low-dose LPS, and high-dose LPS conditions (total of 6 replicates) were utilized, in order to maintain consistency with RNA-seq replicate numbers. No RNA-seq data was excluded and no mutant ChIP-seq data was excluded.
Replication	Replication was successful
Randomization	No randomization was required as there were no covariates.
Blinding	Investigators were not blinded to the genotype or LPS-dosage, as blinding was not relevant to our statistical methods. There were no subjective analyses included.

Reporting for specific materials, systems and methods

We require information from authors about some types of materials, experimental systems and methods used in many studies. Here, indicate whether each material, system or method listed is relevant to your study. If you are not sure if a list item applies to your research, read the appropriate section before selecting a response.

Materials & experimental systems

n/a	Involved in the study	
☐	☒	Antibodies
☒	☐	Eukaryotic cell lines
☒	☐	Palaeontology and archaeology
☐	☒	Animals and other organisms
☒	☐	Human research participants
☒	☐	Clinical data
☒	☐	Dual use research of concern

Methods

n/a	Involved in the study	
☐	☒	ChIP-seq
☐	☒	Flow cytometry
☒	☐	MRI-based neuroimaging

Antibodies

Antibodies used	anti-H3K27ac, abcam, cat: ab4729, lot: GR312651-2
Validation	All the validation documents are available on the manufacturer's websites. We did not conduct further validation.

Animals and other organisms

Policy information about studies involving animals; ARRIVE guidelines recommended for reporting animal research

Laboratory animals	8-12 week old male C57/BL6 mice
Wild animals	The study did not involve wild animals
Field-collected samples	The study did not involve field collected samples
Ethics oversight	Virginia Tech's Institutional Animal Care and Use Committee (IACUC)

Note that full information on the approval of the study protocol must also be provided in the manuscript.

ChIP-seq

Data deposition

☒ Confirm that both raw and final processed data have been deposited in a public database such as GEO.

☒ Confirm that you have deposited or provided access to graph files (e.g. BED files) for the called peaks.

Data access links
May remain private before publication.

https://www.ncbi.nlm.nih.gov/geo/query/acc.cgi?acc=GSE168190

Files in database submission

WT-PBS-1_H3K27ac.fastq
WT-PBS-2_H3K27ac.fastq
WT-Low-1_H3K27ac.fastq
WT-Low-2_H3K27ac.fastq
WT-High-1_H3K27ac.fastq
WT-High-2_H3K27ac.fastq
WT-PBS_input.fastq
WT-Low_input.fastq
WT-High_input.fastq
KO-TRAM-PBS-1_H3K27ac.fastq
KO-TRAM-PBS-2_H3K27ac.fastq
KO-TRAM-Low-1_H3K27ac.fastq
KO-TRAM-Low-2_H3K27ac.fastq
KO-TRAM-High-1_H3K27ac.fastq
KO-TRAM-High-2_H3K27ac.fastq
KO-TRAM-PBS_input.fastq
KO-TRAM-Low_input.fastq
KO-TRAM-High_input.fastq
KO-IRAKM-PBS-1_H3K27ac.fastq
KO-IRAKM-PBS-2_H3K27ac.fastq
KO-IRAKM-Low-1_H3K27ac.fastq
KO-IRAKM-Low-2_H3K27ac.fastq
KO-IRAKM-High-1_H3K27ac.fastq
KO-IRAKM-High-2_H3K27ac.fastq
KO-IRAKM-PBS_input.fastq
KO-IRAKM-Low_input.fastq
KO-IRAKM-High_input.fastq

WT-PBS-1_RNA.fastq
WT-PBS-2_RNA.fastq
WT-Low-1_RNA.fastq
WT-Low-2_RNA.fastq
WT-High-1_RNA.fastq
WT-High-2_RNA.fastq
KO-TRAM-PBS-1_RNA.fastq
KO-TRAM-PBS-2_RNA.fastq
KO-TRAM-Low-1_RNA.fastq
KO-TRAM-Low-2_RNA.fastq
KO-TRAM-High-1_RNA.fastq
KO-TRAM-High-2_RNA.fastq
KO-IRAKM-PBS-1_RNA.fastq
KO-IRAKM-PBS-2_RNA.fastq
KO-IRAKM-Low-1_RNA.fastq
KO-IRAKM-Low-2_RNA.fastq
KO-IRAKM-High-1_RNA.fastq
KO-IRAKM-High-2_RNA.fastq

WT-PBS-1_H3K27ac.bw

WT-PBS-2_H3K27ac.bw
WT-Low-1_H3K27ac.bw
WT-Low-2_H3K27ac.bw
WT-High-1_H3K27ac.bw
WT-High-2_H3K27ac.bw
KO-TRAM-PBS-1_H3K27ac.bw
KO-TRAM-PBS-2_H3K27ac.bw
KO-TRAM-Low-1_H3K27ac.bw
KO-TRAM-Low-2_H3K27ac.bw
KO-TRAM-High-1_H3K27ac.bw
KO-TRAM-High-2_H3K27ac.bw
KO-IRAKM-PBS-1_H3K27ac.bw
KO-IRAKM-PBS-2_H3K27ac.bw
KO-IRAKM-Low-1_H3K27ac.bw
KO-IRAKM-Low-2_H3K27ac.bw
KO-IRAKM-High-1_H3K27ac.bw
KO-IRAKM-High-2_H3K27ac.bw

WT-PBS-1_RNA.txt
WT-PBS-2_RNA.txt
WT-Low-1_RNA.txt
WT-Low-2_RNA.txt
WT-High-1_RNA.txt
WT-High-2_RNA.txt
KO-TRAM-PBS-1_RNA.txt
KO-TRAM-PBS-2_RNA.txt
KO-TRAM-Low-1_RNA.txt
KO-TRAM-Low-2_RNA.txt
KO-TRAM-High-1_RNA.txt
KO-TRAM-High-2_RNA.txt
KO-IRAKM-PBS-1_RNA.txt
KO-IRAKM-PBS-2_RNA.txt
KO-IRAKM-Low-1_RNA.txt
KO-IRAKM-Low-2_RNA.txt
KO-IRAKM-High-1_RNA.txt
KO-IRAKM-High-2_RNA.txt

Genome browser session
(e.g. UCSC)

Not available

Methodology

Replicates

For each genotype and assay type, BMDMs were isolated from ~6-7 mice (i.e. ~6-7 WT for ChIP-seq, ~6-7 each of WT, TRAM-KO, and IRAK-M for RNA-seq) and pooled, before splitting into three cell culture plates to be stimulated with PBS, low-dose LPS, or High-dose LPS. Two or more technical replicates per plate were assayed and sequenced, with good correlation between technical replicates.

Sequencing depth

WT-PBS-1: Total Reads: 13.8 million, Uniquely Mapped: 12.0 million, Read Length: 50nt, Single End
WT-PBS-2: Total Reads: 15.9 million, Uniquely Mapped: 14.0 million, Read Length: 50nt, Single End
WT-Low-1: Total Reads: 21.4 million, Uniquely Mapped: 18.2 million, Read Length: 50nt, Single End
WT-Low-2: Total Reads: 19.4 million, Uniquely Mapped: 16.8 million, Read Length: 50nt, Single End
WT-High-1: Total Reads: 19.4 million, Uniquely Mapped: 16.2 million, Read Length: 50nt, Single End
WT-High-2: Total Reads: 21.0 million, Uniquely Mapped: 18.1 million, Read Length: 50nt, Single End
WT-PBS_input: Total Reads: 15.0 million, Uniquely Mapped: 12.4 million, Read Length: 50nt, Single End
WT-Low_input: Total Reads: 15.6 million, Uniquely Mapped: 12.9 million, Read Length: 50nt, Single End
WT-High_input: Total Reads: 15.3 million, Uniquely Mapped: 12.2 million, Read Length: 50nt, Single End

KO-TRAM-PBS-1: Total Reads: 36.1 million, Uniquely Mapped: 25.9 million, Read Length: 50nt, Single End
KO-TRAM-PBS-2: Total Reads: 34.4 million, Uniquely Mapped: 23.5 million, Read Length: 50nt, Single End
KO-TRAM-Low-1: Total Reads: 15.6 million, Uniquely Mapped: 10.5 million, Read Length: 50nt, Single End
KO-TRAM-Low-2: Total Reads: 25.3 million, Uniquely Mapped: 17.9 million, Read Length: 50nt, Single End
KO-TRAM-High-1: Total Reads: 20.2 million, Uniquely Mapped: 13.7 million, Read Length: 50nt, Single End
KO-TRAM-High-2: Total Reads: 21.8 million, Uniquely Mapped: 14.6 million, Read Length: 50nt, Single End
KO-TRAM-PBS_input: Total Reads: 18.3 million, Uniquely Mapped: 15.2 million, Read Length: 50nt, Single End
KO-TRAM-Low_input: Total Reads: 14.5 million, Uniquely Mapped: 12.0 million, Read Length: 50nt, Single End
KO-TRAM-High_input: Total Reads: 16.6 million, Uniquely Mapped: 13.8 million, Read Length: 50nt, Single End

KO-IRAKM-PBS-1: Total Reads: 17.8 million, Uniquely Mapped: 11.1 million, Read Length: 50nt, Single End
KO-IRAKM-PBS-2: Total Reads: 22.7 million, Uniquely Mapped: 13.8 million, Read Length: 50nt, Single End
KO-IRAKM-Low-1: Total Reads: 16.9 million, Uniquely Mapped: 11.2 million, Read Length: 50nt, Single End
KO-IRAKM-Low-2: Total Reads: 37.5 million, Uniquely Mapped: 25.0 million, Read Length: 50nt, Single End
KO-IRAKM-High-1: Total Reads: 48.4 million, Uniquely Mapped: 33.2 million, Read Length: 50nt, Single End
KO-IRAKM-High-2: Total Reads: 28.1 million, Uniquely Mapped: 19.7 million, Read Length: 50nt, Single End
KO-IRAKM-PBS_input: Total Reads: 25.1 million, Uniquely Mapped: 20.3 million, Read Length: 50nt, Single End
KO-IRAKM-Low_input: Total Reads: 32.4 million, Uniquely Mapped: 26.3 million, Read Length: 50nt, Single End
KO-IRAKM-High_input: Total Reads: 37.1 million, Uniquely Mapped: 30.3 million, Read Length: 50nt, Single End

Antibodies	anti-H3K27ac, abcam, cat: ab4729, lot: GR312651-2; APC/Cy7 anti-mouse/humanC D11b antibody (BioLegend, cat: 101226); FITC anti-mouse Ly-6C antibody (BioLegend, cat: 128006); AF647 anti-mouse S100A8 antibody (NOVUS, cat: NBP2-27067AF647).
Peak calling parameters	Read mapping (mm10): bowtie -t -p 16 -S /path/to/mm10/index ChIPorInput.fastq -S ChIPorInput.sam<br>Peak calling: macs2 callpeak -t ChIP.bed -c Input.bed -f BED -g mm -n SampleName -q 0.05
Data quality	WT-PBS-1: 25,727<br>WT-PBS-2: 31,536<br>WT-Low-1: 25,820<br>WT-Low-2: 22,316<br>WT-High-1: 15,348<br>WT-High-2: 18,204<br><br>KO-TRAM-PBS-1: 22,255<br>KO-TRAM-PBS-2: 29,432<br>KO-TRAM-Low-1: 34,552<br>KO-TRAM-Low-2: 28,274<br>KO-TRAM-High-1: 41,880<br>KO-TRAM-High-1: 45,166<br><br>KO-IRAKM-PBS-1: 22,651<br>KO-IRAKM-PBS-2: 34,861<br>KO-IRAKM-Low-1: 28,487<br>KO-IRAKM-Low-2: 27,880<br>KO-IRAKM-High-1: 38,542<br>KO-IRAKM-High-2: 20,557
Software	Trim Galore!: 0.4.1<br>fetchChromSizes: 332<br>bowtie: 1.1.2<br>bedtools: 2.26.0<br>samtools: 1.9<br>bedGraphToBigWig : 332<br>bedSort: 332<br>MACS2: 2.1.1.20160309<br>cor (stats): 3.6.0<br>ggplot2: 3.3.1<br>HOMER: 4.10.3<br>clusterProfiler: 3.18.0<br>Salmon: 1.2.1<br>IsoformSwitchAnalyzeR: 3.12<br>CPAT<br>SignalP: 5.0<br>Pfam (HMMER): 2.41.1<br>NetSurfP2.0

Flow Cytometry

Plots

Confirm that:

☒ The axis labels state the marker and fluorochrome used (e.g. CD4-FITC).

☒ The axis scales are clearly visible. Include numbers along axes only for bottom left plot of group (a 'group' is an analysis of identical markers).

☒ All plots are contour plots with outliers or pseudocolor plots.

☒ A numerical value for number of cells or percentage (with statistics) is provided.

Methodology

Sample preparation	Crude BM cells were isolated from the mice and cultured as previously published. After treatment, the cells were harvested, rinsed with PBS and filtered through a 70 μm strainer to prepare single cell suspension. The cells were incubated with anti-CD16/-CD32 antibodies (BD Biosciences, no. 553141) to block Fc-receptors. For detecting surface phenotype, the cells were stained with anti-CD11b and anti-Ly6C antibodies, and PI was added before flow cytometry. For detecting S100A8 expression, the cells were fixed, permeabilized, stained with anti-S100Ab antibody, and then analyzed by flow cytometry.
Instrument	FACSCantoII
Software	FlowJo v10
Cell population abundance	The frequencies of cells are stated in the representative images and quantifications.

Gating strategy	The cells were gated by FSC and PI. Dead cells were discriminated, and live cells were analyzed for the surface expression of CD11b and Ly6C.

☒ Tick this box to confirm that a figure exemplifying the gating strategy is provided in the Supplementary Information.
